# Effects of E-Liquids and Their Aerosols with and Without Flavors on OKF6/TERT-2 Oral Epithelial Cells: Consequences of Heating and Aerosolization on Cell Viability

**DOI:** 10.3390/dj14100656

**Published:** 2026-10-09

**Authors:** Dominic L. Palazzolo, Maleiya Hill, Sumar Beauti, Mikaela Brown, Avni Patel, Angela Mamari, Giancarlo A. Cuadra

**Affiliations:** 1 Department of Physiology, College of Osteopathic Medicine, Lincoln Memorial University-DeBusk, Harrogate, TN 37752, USA; maleiya.hill@lmunet.edu (M.H.); sumar.beauti@lmunet.edu (S.B.); mikaela.brown@lmunet.edu (M.B.); avni.patel@lmunet.edu (A.P.); 2 Department of Biology, Muhlenberg College, Allentown, PA 18104, USA; amamari@muhlenberg.edu (A.M.); giancarlocuadra@muhlenberg.edu (G.A.C.)

**Keywords:** oral epithelium, OKF6/TERT-2 cells, flavored E-liquids, heating and aerosolization, cell morphology, viability, cytotoxicity

## Abstract

**Background:** Multiple effects of flavored E-liquids or E-liquid aerosols on various cell lines have been reported. However, concentrations of E-liquids and aerosols are not usually equivalent, making it difficult to determine the relative effect heating and aerosolization plays on the cells tested. To match concentrations of E-liquids with their aerosols, the mass and the concentration of the E-liquid nicotine dissolved in culture media must be compared to that of the aerosol bubbled into the culture media. The aim of this study is to compare the effects of equivalent amounts of E-liquids and their aerosols ± flavors (tobacco, menthol, cinnamon, strawberry and blueberry) on the morphology and viability of OKF6/TERT-2 oral epithelial cells. **Methods:** HPLC-UV was used to measure nicotine concentrations in culture media containing E-liquids or aerosols. Morphology and viability of OKF6/TERT-2 cells exposed to equivalent amounts of E-liquids or aerosols over a 24 h period were subsequently assessed using light microscopy, LDH cytotoxicity assays, flow cytometry and DAPI staining. **Results:** One hundred puffs of aerosol bubbled into 50 mL of media is equivalent to 0.17% E-liquid. As indicated by altered morphology, increased LDH activity, flow cytometry and DAPI fluorescent staining, OKF6/TERT-2 cells are negatively impacted by 100 puffs of aerosols flavored with strawberry resulting in reduced cell viability, while the same amount of E-liquids directly administered have minimal to no effect. **Conclusions:** The data suggests that heating and aerosolization enhance the cytotoxic effect of strawberry flavored E-liquid.

## 1. Introduction

Electronic nicotine delivery systems (ENDSs), commonly known as electronic cigarettes (ECIGs) or vapes, have rapidly transformed from smoking cessation aids into a widespread social and public health phenomenon [1,2,3]. Initially developed as an alternative to conventional tobacco products, vaping has grown into a global, multibillion-dollar market that especially appeals to adolescents and young adults [4]. This rise in popularity is accompanied by growing concerns about its potential health consequences. Despite promotion as a “safer” option than smoking, the true impact of inhaling ECIG aerosols on human tissues remains uncertain and under active investigation [5,6].

The most basic ECIG device consists of a battery, a heating element, and a cartridge or reservoir containing a liquid (henceforth referred to as E-liquid). When the device is activated, the heating element vaporizes the E-liquid’, usually a mixture of propylene glycol (PG), vegetable glycerol (VG), nicotine, and a variety of flavoring chemicals, to create an aerosol that is inhaled or vaped by the user. Nicotine content varies across products and E-liquids contain flavoring additives that make vaping more appealing, especially to young adults [7,8]. These flavoring compounds, while approved for ingestion in food, are not necessarily safe for inhalation and may produce harmful byproducts when heated and aerosolized [9,10,11,12]. Thermal degradation of E-liquid could possibly alter its chemical composition making an otherwise innocuous E-liquid into one that is cytotoxic [13]. Tobacco-specific nitrosamines (TSNAs) and volatile organic compounds (VOCs) have been detected in ECIG aerosols, albeit at extremely low levels [14,15,16]. Similarly, Williams et al. (2019) [17] report the presence of metals in ECIG aerosols presumably originating from the heating coils or filaments within the device. Heating the base humectants (i.e., PG and/or VG) is known to produce harmful toxins. The production of deleterious carbonyls and reactive oxygen species (ROS) in the aerosol directly correlates with increasing power output of the ENDS and hence can negatively impact cell viability [18]. Mercier et al. (2024) [19] report that E-liquid aerosols with a higher PG/VG ratios induce more cytotoxicity in human lung epithelial cells, indicating that PG in the E-liquid is the primary determinant of cytotoxicity. Despite the high ratio of PG/VG inducing cytotoxicity, the mechanism of cytotoxicity does not appear to involve inflammation and/or oxidative stress since interleukin (IL)-8 and 8-hydroxy-2-deoxyguanosine (8-OHdG) levels, respectively, were comparable to controls [19].

Much research focuses on assessing how E-liquids ± flavors (or their aerosols) affect cytotoxicity and viability of human cells. The addition of flavoring agents to the E-liquid concoction further increases the possibility of generating additional toxins from the heating and aerosolization process. For example, Behar et al. (2018) [20], using the A549 lung epithelial cell line, found conflicting results within the same study in terms of cytotoxicity between E-liquids and aerosols. In another study [21], osteoblast-like cells generated more ROS when exposed to un-vaped cinnamon flavored E-liquid than its aerosol counterpart. Rowell et al. (2017) [22] reported similar dose-dependent reductions in the viability of airway epithelial cells between direct administration of E-liquids (0% to 3%) and the addition of captured aerosols (0 to 35 puffs) to the cell media. From these studies, it is evident that aerosols represent a different type of exposure due to heating and vaporization of the E-liquid. The addition of flavors to the E-liquids further complicates these differences. Puff topography, ECIG device and vape parameters (i.e., coil resistance, voltage, wattage and temperature) can amplify or depress these differences. Standardizing experimental exposure methods, such as determining equivalent concentrations of aerosolized and non-aerosolized E-liquid is key in understanding vaping-related toxicity.

The oral cavity is the first site of contact of inhaled aerosols making it vulnerable to potential damage induced by vaping. Therefore, OKF6/TERT-2 cells [23], an immortalized oral epithelial cell line, serves as a model to examine vape-induced consequences on the oral epithelium. To fully appreciate the effect that heating and aerosolization have on E-liquids, the concentrations of aerosolized and non-aerosolized E-liquid must be equated. From weight and volume of E-liquid aerosolized and the original concentration of nicotine in the E-liquid before aerosolization, the nicotine concentration of aerosolized E-liquid bubbled into culture media can be determined and hence the concentration of aerosolized E-liquid, assuming the aerosolization of nicotine and other E-liquid components occurs at the same rate. This critical assumption is supported by Noel and Gosh (2022) [24] who report that nicotine concentrations from E-liquids containing strawberry, vanilla or catalan cream flavors and their aerosolized counterparts do not differ significantly. On the other hand, they observed significant variations in generated carbonyls between these same flavors. In another report, Khachtootian et al. (2022) [25] found that the transfer efficiencies of several flavor chemicals (from machine-vaped refill fluid to aerosol) depend on the physical properties of the chemicals, ECIG puffing parameters, and puff topography, but that transfer efficiency of nicotine remained consistent and was not affected by these factors. Regardless of whether or not nicotine aerosolization is altered by various flavoring agents, aerosolized nicotine can potentially alter the chemistry of flavor chemicals into more harmful products. As seen by Kerber et al. (2022) [26], E-liquids without flavoring agents produce toxic chemicals on aerosolization. The addition of flavorants such as benzaldehyde, vanillin, benzyl alcohol, and trans-cinnamaldehyde to the base E-liquid further increases the production of these chemicals. Interestingly, the addition of nicotine to the flavored E-liquid results in further increase or reduction in the production of these toxic compounds. This indicates that the interaction of flavoring agents with nicotine during the aerosolization process is complex and requires further investigation which is beyond the scope of this study.

Assessing how E-liquids and ECIG-generated aerosols influence OKF6/TERT-2 cell morphology, viability and DNA content will provide valuable insight into the biological risks associated with vaping. Using nicotine as a marker, this study aims to compare the effects of equivalent amounts of non-aerosolized E-liquids ± flavors with aerosolized E-liquids bubbled into the media. We hypothesize that heating and aerosolization of E-liquids, as compared to E-liquids per se, will have a greater effect on the morphology, viability and DNA content of OKF6/TERT-2 cells, at least for some flavored E-liquids.

## 2. Materials and Methods

### 2.1. Reagents and Supplies

Laboratory materials and reagents were obtained from ThermoFisher Scientific (Waltham, MA, USA) unless otherwise noted.

### 2.2. Culture Media

Keratinocyte Serum-Free Medium (KSFM) was prepared with 30 μg/mL of bovine pituitary extract (BPE), 3 ng/mL epithelial growth factor, 0.3 mM calcium chloride, 2 mM glutamine, and 100 U/mL penicillin and streptomycin. Dulbecco’s Modified Eagle’s Medium/Ham’s Nutrient Mixture F-12 (DMEM/F12) was supplemented with the same nutrients added to KSFM [27,28]. A 1:1 *v*/*v* mixture of prepared DMEM/F12 and KSFM is from here on referred to as DFK. The rationale for DFK use is two-fold: first, cells grow confluent quickly, which saves time and effort; and second, DMEM/F12 is more cost effective. Using oral epithelial cells, we have previously compared KSFM and DFK culture media for any potential differences in gene expression and found none [27,28].

### 2.3. E-Liquids, Vaping Device and Aerosol Generation

**Figure 1:**
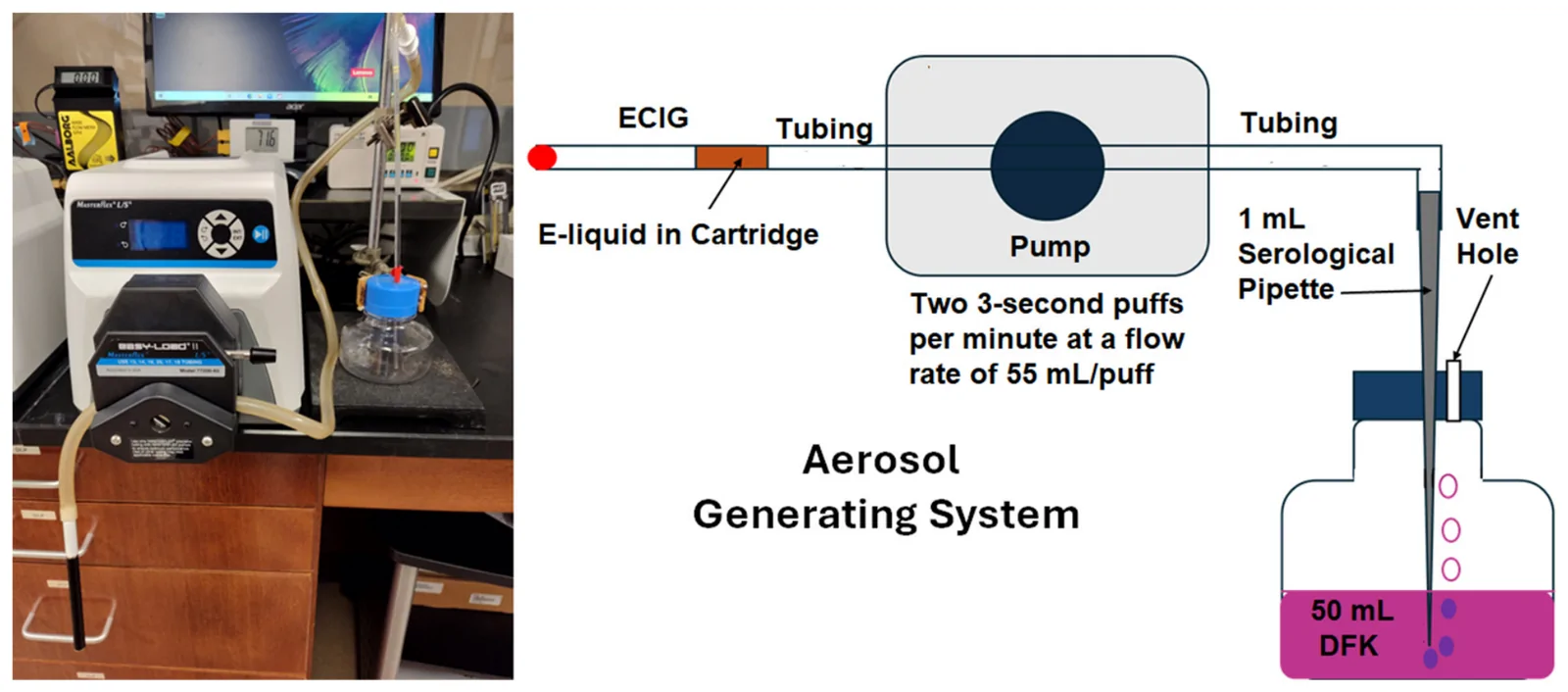
Depiction of the aerosol generating system, where aerosol is bubbled into 50 mL of DFK.

E-liquid was composed of 50% propylene glycol and 50% vegetable glycerine (aka glycerol) with 20 mg/mL of 99% (S)-(-)-nicotine (Alfa Aesar, Tewksbury, MA, USA) and is subsequently referred to as Flavorless E-liquid. Alternatively, this base E-liquid was supplemented with 5% concentrated flavoring agents (either tobacco, menthol, cinnamon, strawberry or blueberry) purchased online from Liquid Nicotine Wholesalers (Phoenix, AZ, USA). The E-liquids were aerosolized using Apollo E-cigarette (Apollo Future Technology, Inc, Las Vegas, NV, USA) [29,30] consisting of a battery (≈4.2 V/250 mAh), which lasts for ≈200 puffs when fully charged, and a blank cartridge (with resistance coil ≈ 2.0 Ω and power output of ≈8.8 W). The blank cartridges are filled with ≈1 mL of E-liquid ± flavoring agents. This “cig-a-like” device is self-actuated by negative pressure created whenever the aerosolization pumps are turned on. The aerosolization system consists of a Masterflex model 77200-60 L/S peristaltic pump (Vernon Hills, IL, USA) used to transport aerosol from the ECIG device and through 100 cm of Masterflex Tygon S3^®^ B-44-4X (ThermoFisher Scientific, Waltham, MA, USA) precision tubing (ID = 8.0 mm, 1.6 mm wall thickness). The opposite end of this tubing connects to a 1 mL serological pipette which conveys aerosol into 50 mL of DFK culture media within closed but vented 125 mL plastic media bottles as shown in Figure 1 [31]. Before the start of each aerosolization run, the ECIG batteries were fully charged to ensure maximum power output. In addition, fresh blank ECIG cartridges were used for every aerosolization run to make certain that each resistance coil was exposed to only one flavor and operated at stated specification. The serological pipettes and media bottles were prepackaged as sterile, and all pump tubing sterilized before use in every aerosolization run. Puff topography of generated aerosol follows Cooperation Centre for Scientific Research Relative to Tobacco (CORESTA) guidelines of Method No. 81 (i.e., two 3 s puffs per minute with flow rate of 55 mL/puff) [32]. Vaping parameters are outlined in Supplementary Table S1. In addition, the outside temperature of the ECIG cartridge remained consistent for up to 60 min of continuous puffing as shown in Supplementary Figure S1.

### 2.4. High-Performance Liquid Chromatography (HPLC) for Nicotine Quantification

Standard solutions of nicotine were prepared in DFK media at concentrations of 125.0, 62.5, 31.3, 15.6 and 7.8 µg/mL. Standards and samples of DFK exposed to 25, 50, or 100 puffs of E-liquid ± flavored aerosols were analyzed by HPLC coupled with photodiode array detection, as previously described [31,33]. A Shimadzu HPLC system (Columbia, MD, USA) was used to quantitate nicotine and included the following: a photodiode array detector (SPD-M20A), a pump (LC-20AT), a column oven (CTO-20A), an in-line membrane degasser (DGU-20A3R), and a Rheodyne 7725I (Rohnert Park, CA, USA) manual injector with a 20 µL loop (30 µL injection volume). Nicotine was eluted on a Phenomenex (Torrance, CA, USA) 15 cm, Kinetex^®^ 5 µm reversed-phase C-18 column. The column temperature was maintained at 35 °C. Nicotine was detected at ultraviolet wavelengths between 230 and 300 nm, and quantification was carried out at 260 nm. The mobile phase was isocratically delivered at a rate of 1.2 mL/min. The mobile phase consisted of 10% acetonitrile in 20 mM ammonium formate adjusted to pH 8.5 with 50% ammonium hydroxide. Chromatographic parameters were computer-controlled using a Shimadzu Lab Solutions workstation (Columbia, MD, USA). Nicotine concentrations were determined from 50 mL of DFK growth media containing 1% E-liquid or DFK media (50 mL) following exposure to 25, 50, and 100 puffs of ECIG-generated aerosol ± flavors.

### 2.5. Determination of Weight, Volume and Percent Recovery of ECIG-Generated Aerosol

Before starting the aerosolization process, blank cartridges were filled with flavorless E-liquid and weighed. Following delivery of 25, 50 or 100 puffs of flavorless aerosol into the DFK media, the cartridges were reweighed to determine the weights of the aerosolized E-liquid. Assuming that the specific density of flavorless E-liquid is 1.18 g/mL, as determined from a previous study [34], the volumes of aerosolized E-liquid after 25, 50 or 100 puffs were also determined. From HPLC quantifications of nicotine bubbled into DFK and given the initial concentration of nicotine in E-liquid as 20 mg/mL, the percent recoveries of nicotine in DFK were calculated. If the aerosolization process is uniform for all components of the E-liquid, we can assume the percent recovery of nicotine is equivalent to the percent recovery of the other E-liquid components. In another set of experiments, the contents of nicotine following exposure of 100 puffs of aerosol ± flavors bubbled into 50 mL of DFK were determined by HPLC, along with their respective percent recoveries. Finally, to determine if heating and subsequent aerosolization of E-liquid influences the recovery of nicotine, the concentrations of nicotine following exposure to 100 puffs of aerosol ± flavors bubbled into 50 mL of DFK were compared to an equivalent amount of E-liquid ± flavors directly added to the 50 mL of DFK.

### 2.6. Morphology of OKF6/TERT-2 Cells

OKF6/TERT-2 cells were generously provided by Dr. Gill Diamond from Louisville University, School of Dentistry, but the cell line was originally established by Dickson et al. [23]. Growth and expansion of OKF6/TERT-2 cells is described in detail in Cuadra et al. (2023) [27]. Briefly, for the first 24 h, these cells were cultured in flat-bottom 24-well plates containing 1 mL KSFM (as specified above) under standard conditions (37 °C, 5% CO_2_). The next day, KSFM was removed from the cell culture and replaced with 1 mL of DFK, which bathed the OKF6/TERT-2 until they became 90+% confluent, usually three to four days. Once confluent, the DFK media was removed and replaced with 1 mL of DFK exposed to 100 puffs of E-liquid aerosol ± flavors, or an equivalent amount of non-aerosolized E-liquid (as determined from percent recoveries) and allowed to incubate at 37 °C, 5% CO_2_ for an additional 24 h. Twenty-four hours post-treatments, images of OKF6/TERT-2 cell monolayers were taken using an AMG EVOS XL core microscope (ThermoFisher, Waltham, MA, USA) equipped with an integrated camera. After imaging, the supernatants were collected and stored at −80 °C for subsequent LDH activity assays.

### 2.7. Viability of OKF6/TERT-2 Cells

#### 2.7.1. LDH Cytotoxicity Assay

The Invitrogen CyQUANT (Carlsbad, CA, USA) Lactate dehydrogenase (LDH) cytotoxicity assay kit (Cat. No. C20300) was used according to manufacturer’s instructions as an index of cell viability [35]. Briefly, untreated OKF6/TERT-2 cell monolayers were lysed with a lysis buffer (provided with kit) and were used to reference 100% LDH activity (an index of 0% viability). At the time of the assay, 50 µL of thawed supernatants were added to 50 µL of the reaction mixture (provided with kit) and incubated for 30 min in the dark at room temperature. After incubation, 50 µL of the stopping solution (provided with kit) was added to all reactions, and the absorbance was read at 490 and 680 nm. The data was normalized by subtracting the OD680 nm results from the OD490 nm results (OD_490nm_ − OD_680nm_) to remove any background interference.

#### 2.7.2. Flow Cytometry

**Figure 2:**
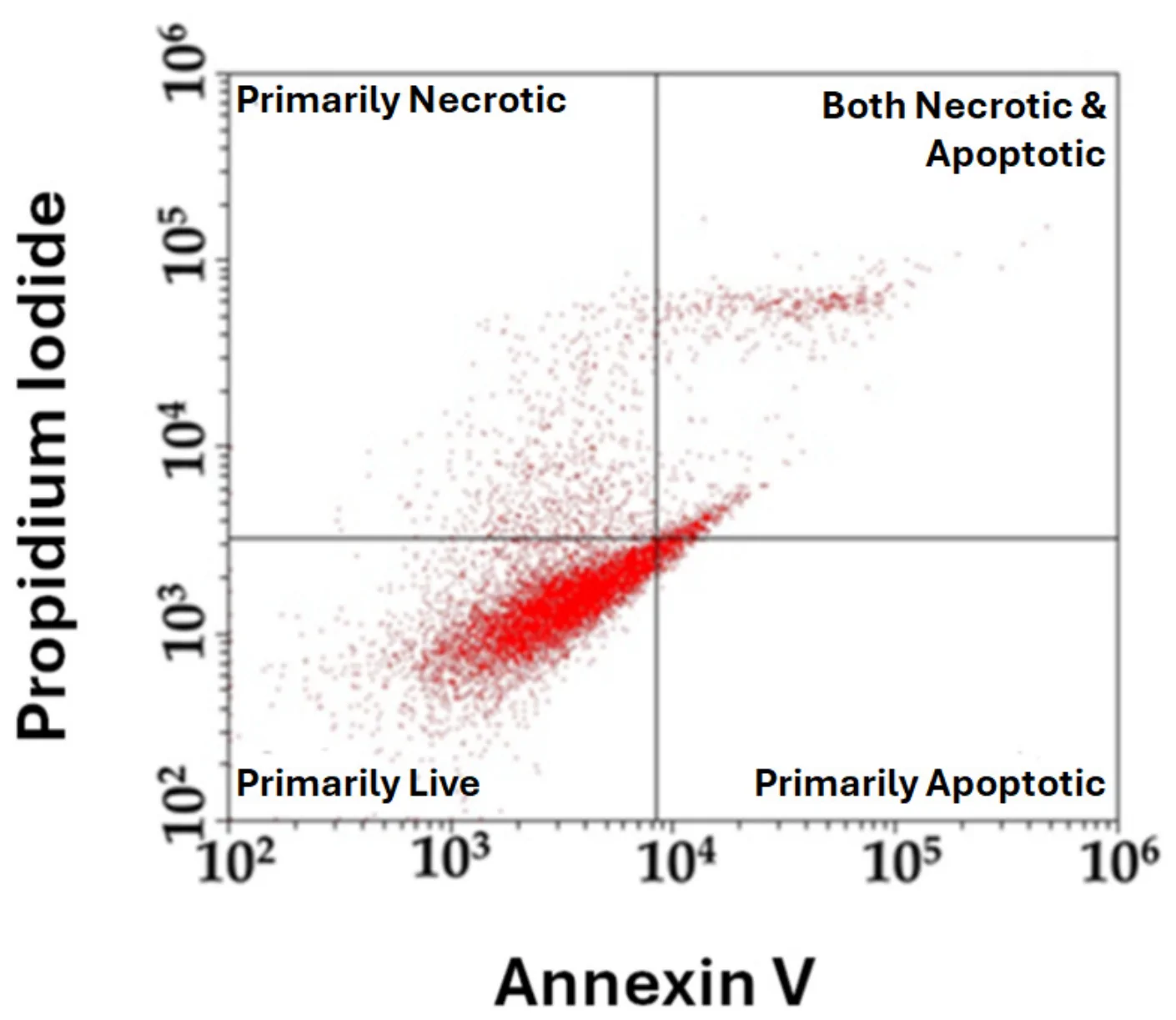
Depiction of a representative flow cytometry dot plot where gating using unstained controls divides cell populations into quadrants based on the annexin V and PI fluorescence for individual runs. Lower left quadrant reflects primarily live cells. Upper left and lower right quadrants represent cells that are primarily necrotic and apoptotic, respectively. Upper right quadrant shows cells that are both necrotic and apoptotic.

Flow cytometry was used to assess viability by necrosis/apoptosis of OKF6/TERT-2 cells following treatment with E-liquids flavors and their corresponding aerosols. Cells were cultured and treated as described in Section 2.6. After 24 h of treatment, cells were harvested and stained using the Dead Cell Apoptosis kit (Cat. No. 501137620), according to manufacturer’s instructions [36]. Briefly, cells were washed with PBS, resuspended in 1× annexin V binding buffer and stained with Annexin V Alexa Fluor™ 488 and propidium iodide (PI) provided in the kit. Stained cells were incubated at room temperature and analyzed by flow cytometry using 488 nm excitation for annexin V and 535 nm excitation for PI. Fluorescence emission was collected at 530 and 575 nm for annexin V and PI, respectively, to distinguish viable, necrotic, and apoptotic populations. Gating was determined based on unstained controls. Figure 2 depicts a representative flow cytometry dot plot where annexin V-stained cells ranging from 10^2^ to 10^6^ are shown on the *x*-axis, while propidium iodide-stained cells ranging from 10^2^ to 10^6^ are shown on the *y*-axis. The gating generated the quadrants indicating the threshold or boundary settings used to divide populations based on viability status.

#### 2.7.3. DAPI Staining and Fluorescent Microscopy

Sterile 12 mm Bellco Glass coverslips (ThermoFisher, Waltham, MA, USA; catalog number 50-194-4702) were treated with 10% poly-D-Lysine in PBS for 10 min at room temperature and then placed in the bottom of sterile 12-well plates. OKF6/TERT-2 cells grown to confluency in a T-25 flask and were detached from the flask using TrypLE^®^. Cells were pelleted and resuspended in 5 mL of KSFM. Fifty microliters of OKF6/TERT-2 cells were grown directly on the coverslips. Growth, expansion and treatment of cells were performed as described in Section 2.6 above. Media were removed from all wells; cells were washed with 1 mL of PBS and fixed with 4% paraformaldehyde for ten minutes. Next, the paraformaldehyde was removed from all wells, cells were washed with 1 mL PBS, and permeabilized with 0.1% TritonX-100 for ten minutes. In the final step, the 0.1% TritonX-100 was removed, cells were washed with 1 mL of PBS, and the coverslips were lifted from the wells and mounted on glass microscope slides with Fluoromount-G Mounting Medium, with DAPI (4′,6-diamidino-2-phenylindole; Cat. No. 00495952; ThermoFisher Scientific, Waltham, MA, USA), a fluorescent stain that binds strongly to adenine–thymine-rich regions in DNA. The OKF6/TERT-2 cells were then observed with a Zeiss AXIO observer Z1 inverted microscope (Oberkochen, Germany) equipped with a Moticam 1080 HDMI and USB camera (Motic^®^, Richmond, BC, Canada) using ultraviolet light as the excitation for DAPI blue (excitation 350 nm, emission 455 nm). Photographic images were taken at 400× total magnification (resolution = 1920 × 1080 pixels) and converted to 8-bit black and white (B&W) images using the public domain software ImageJ 1.53t (National Institute of Health). ImageJ was used to quantify the percent area occupied by DNA, which is indicated by the white area within each B&W image [37,38].

### 2.8. Statistical Analysis

All data is reported as means ± standard error of the mean (SEM) and all comparisons were made using one-way or two-way ANOVA followed by Bonferroni multiple comparison test. Linear regression analysis was used to calculate standard curve statistics and to verify linearity of the weight and volume of E-liquid aerosolized from 25 to 100 puffs. Statistical significance is achieved when *p* < 0.05. Version 5 of Prism (GraphPad Software, San Diego, CA, USA) was used to generate all graphs and perform all statistical tests. All tabulated experimental data and statistical analyses can be found in Supplemental Table S1.

## 3. Results

### 3.1. Nicotine Standard Curve

**Figure 3:**
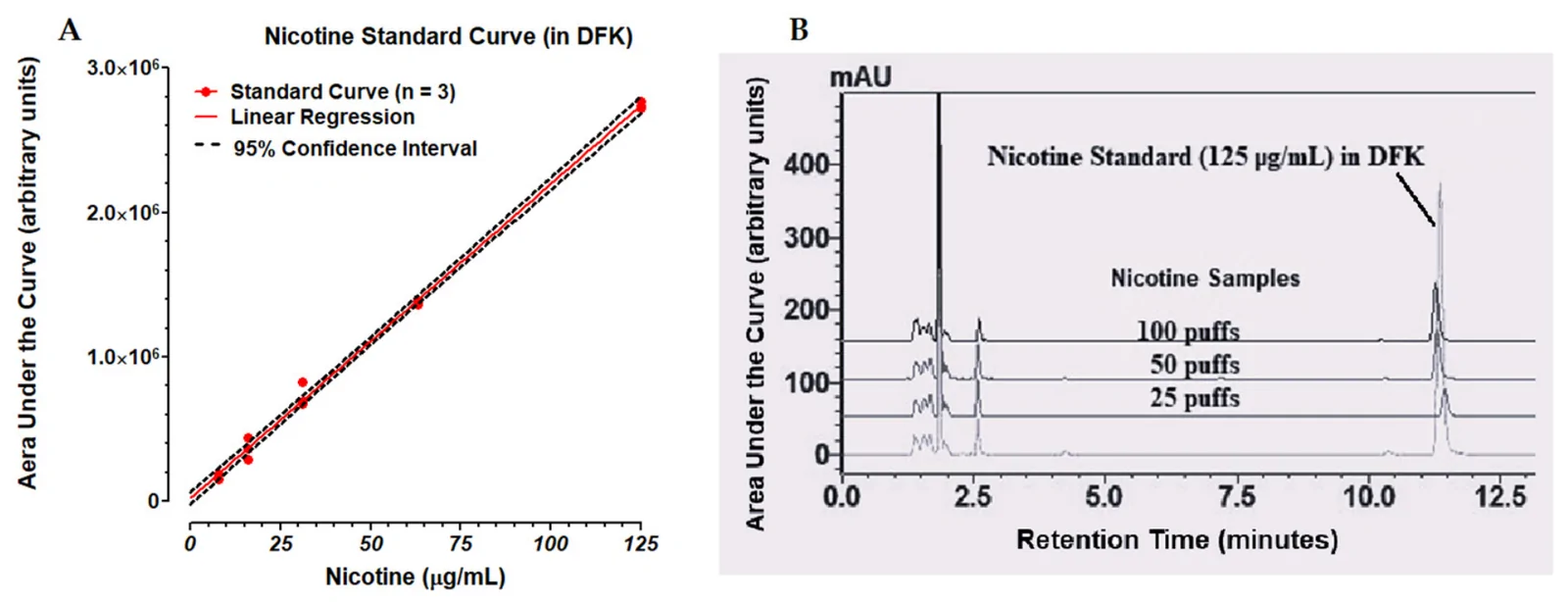
Linear regression of nicotine standard curve (**A**). Red dots represent triplicate values for nicotine standards at 7.8, 15.6, 31.3, 62.5 and 125.0 µg/mL. The red line depicts the linear regression where R^2^ = 0.9984 and the straight-line equation is Y = 21,756 X + 15,283. The black dashed lines define the 95% confidence interval. Chromatographic tracings (**B**) of representative nicotine standards (125 µg/mL) in DFK superimposed over a stack of nicotine samples in DFK that were exposed to 25, 50 or 100 puffs of ECIG-generated flavorless aerosol.

As shown in Figure 3A, HPLC analysis reveals that the nicotine standard curve is linear (R^2^ = 0.9984), in the range of 7.8 to 125.0 µg/mL. Figure 3B illustrates typical standard and sample tracings of nicotine chromatography. Nicotine eluted at retention times ranging from 11.25 to 13.5 min. Linear regression data and statistics are reported in Table 1 and in Supplementary Table S1.

### 3.2. Weight, Volume and Percent Recovery of ECIG-Generated Aerosol

**Figure 4:**
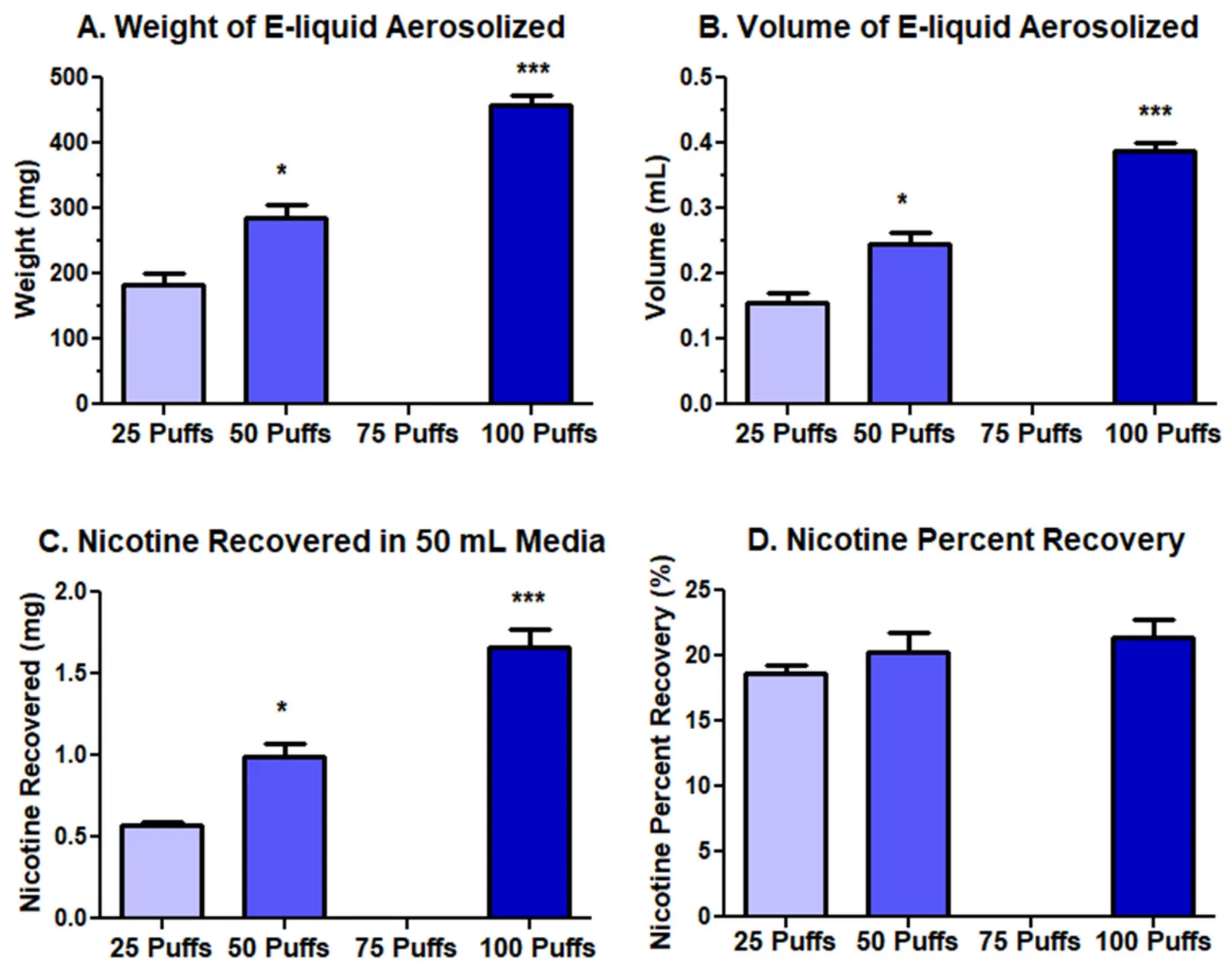
The weight (**A**), and volume (**B**) of nicotine recovered in 50 mL of DFK (**C**) and the percent recovery of nicotine (**D**) from flavorless E-liquid aerosolized following 25, 50 or 100 puffs bubbled into DFK media. Each bar represents the mean ± SEM where *n* = 3 independent samples. * = *p* < 0.05 and *** = *p* < 0.001 as compared to 25 puffs using one-way ANOVAs.

From the weights of E-liquid aerosolized after 25, 50 or 100 puffs and the assumed E-liquid specific density (1.18 g/mL) [34], the volumes of the aerosolized E-liquid were determined (see Supplementary Table S1). Figure 4 displays the weight (A), volume (B), nicotine recovered in 50 mL of DFK (C) and nicotine percent recovery (D) of flavorless E-liquid aerosolized following 25, 50 or 100 puffs bubbled into DFK media (see Supplementary Table S1). Linear regression analysis reveals that the weight (Figure 4A) and volume (Figure 4B) of E-liquid aerosolized from 25 to 100 puffs are linear, with R^2^ values of 0.9516 and 0.9487, respectively, suggesting that the ECIG battery is performing at near maximum power even after 100 puffs. The total content of nicotine recovered from 50 mL of DFK media (Figure 4C) bubbled with flavorless aerosols is calculated to be 0.573, 0.996 and 1.660 mg after 25, 50 or 100 puffs, respectively. Furthermore, linear regression analysis indicates that the nicotine content in the E-liquid aerosolized from 25 to 100 puffs is linear, with an R^2^ value of 0.9389. Given that the initial concentration of nicotine in the E-liquid is 20 mg/mL, the total possible nicotine to be aerosolized is 3.083, 4.901 and 7.775 mg after 25, 50 or 100 puffs, respectively. Consequently, the percent recoveries of nicotine (Figure 4D) are 18.6, 20.3, and 21.3% after 25, 50 or 100 puffs, respectively. As expected, the percent recoveries, regardless of puff number, are similar (*p* > 0.05). From this point on, the assumption is made that the percent recovery of nicotine is equivalent to the percent recovery of the E-liquid. The calculations used to determine nicotine (and hence, E-liquid) percent recovery are outlined in Table 2.

### 3.3. Percent Recovery of E-Liquid Aerosols ± Flavors

**Figure 5:**
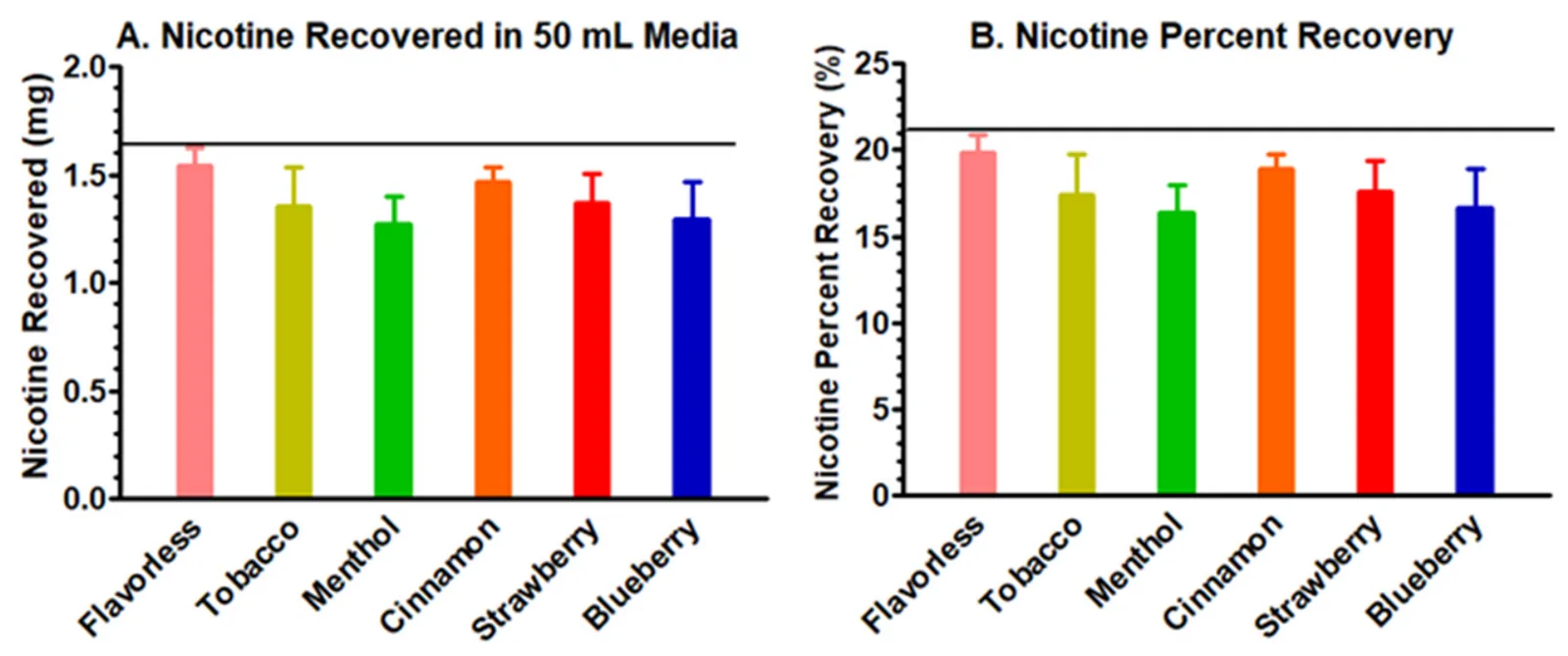
The nicotine recovered (**A**) and the percent recovery of nicotine calculated as actual/theoretical nicotine content (**B**) following exposure to 100 puffs of aerosol ± flavors bubbled into 50 mL of DFK media. Each bar represents the mean ± SEM where *n* = 7 independent samples. Black horizontal lines represent the average amount of nicotine recovered and percent recovery of the flavorless E-liquid after 100 puffs of aerosol were bubbled into 50 mL of DFK media, as shown in Figure 4C,D, respectively. One-way ANOVA reveals no statistical significance.

Figure 5 (and Supplementary Table S1) compares the total amount of nicotine recovered (A) and the nicotine percent recovery (B) after bubbling 100 puffs of aerosol ± flavors into 50 mL of DFK. The actual mean nicotine content recovered between flavor groups ranged between 1.271 and 1.544 mg and is comparable to the nicotine content previously recovered (1.660 mg) in Figure 4C. Similarly, the percent nicotine recoveries (actual/theoretical) between flavor groups ranged from 16.35% to 19.23% and are comparable to the nicotine percent recovery (21.3%) shown in Figure 4D. These results suggest that there is little to no difference in nicotine content recovered and percent recoveries between flavor groups (*p* > 0.05).

### 3.4. Comparison of Nicotine Concentrations in E-Liquids ± Flavors with Their Aerosols in Culture Media

**Figure 6:**
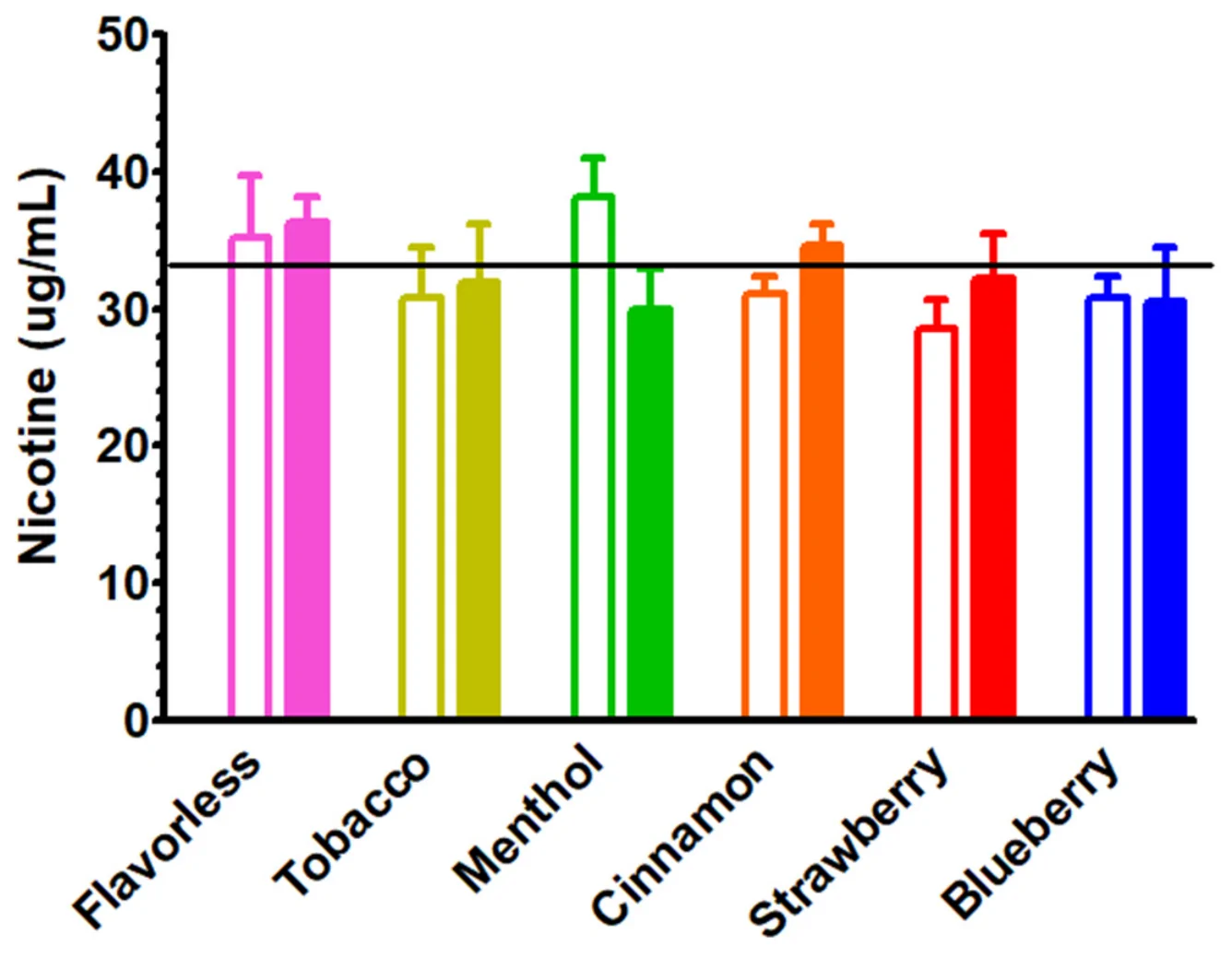
Comparison of nicotine concentrations determined after direct administration of 0.2% E-liquid ± flavors into DFK media (unfilled bars; *n* = 5 independent samples) and 100 puffs of aerosolized E-liquid ± flavors bubbled into DFK media (filled bars; *n* = 7 independent samples) compensated to 0.20%. Each bar represents the mean ± SEM. Black horizontal line represents the average nicotine concentration of the flavorless E-liquid after 100 puffs of aerosol was bubbled into 50 mL of DFK media, as indicated in Table 2. Two-way ANOVA reveals no statistical significance.

Since the volume of flavorless E-liquid aerosolized from 100 puffs is 0.39 mL (Figure 4B) and 21.3% of that volume is recovered in 50 mL of DFK (Figure 4D), the amount of aerosolized E-liquid in the DFK is 0.083 mL, which represents a 0.17% E-liquid solution in DFK (***v*/*v***). The amount of E-liquid directly added to the DFK was rounded up from 0.17% to 0.20%. As seen in Supplementary Table S1, this 0.03% deviation in nicotine concentration did not significantly (*p* > 0.05) alter the nicotine concentration. Furthermore, as indicated in Figure 6, direct administration of 0.20% E-liquid ± flavors (unfilled bars) or 100 puffs of aerosol ± flavors (filled bars) compensated to 0.20% are similar (*p* > 0.05). Direct E-liquid nicotine concentrations ranged between 28.5 and 38.2 µg/mL, while the compensated bubbled aerosol nicotine concentration ranged between 29.9 and 36.3 µg/mL. These results suggest that the amount of 0.20% direct E-liquid and 0.20% of 100 puffs of bubbled aerosol in the DFK culture media are similar, yielding no significant differences (*p* > 0.05 for all comparisons). This suggests that heating and subsequent aerosolization of E-liquid ± flavors have little to no influence on nicotine recoveries in DFK. From this point on, direct addition refers to 0.20% E-liquid ± flavors in DFK while 100 puffs of bubbled aerosol refer to 0.17% E-liquid ± flavors in the DFK culture media.

### 3.5. Effects of E-Liquids ± Flavors and Their Aerosols in Culture Media on OKF6/TERT-2 Cells

**Figure 7:**
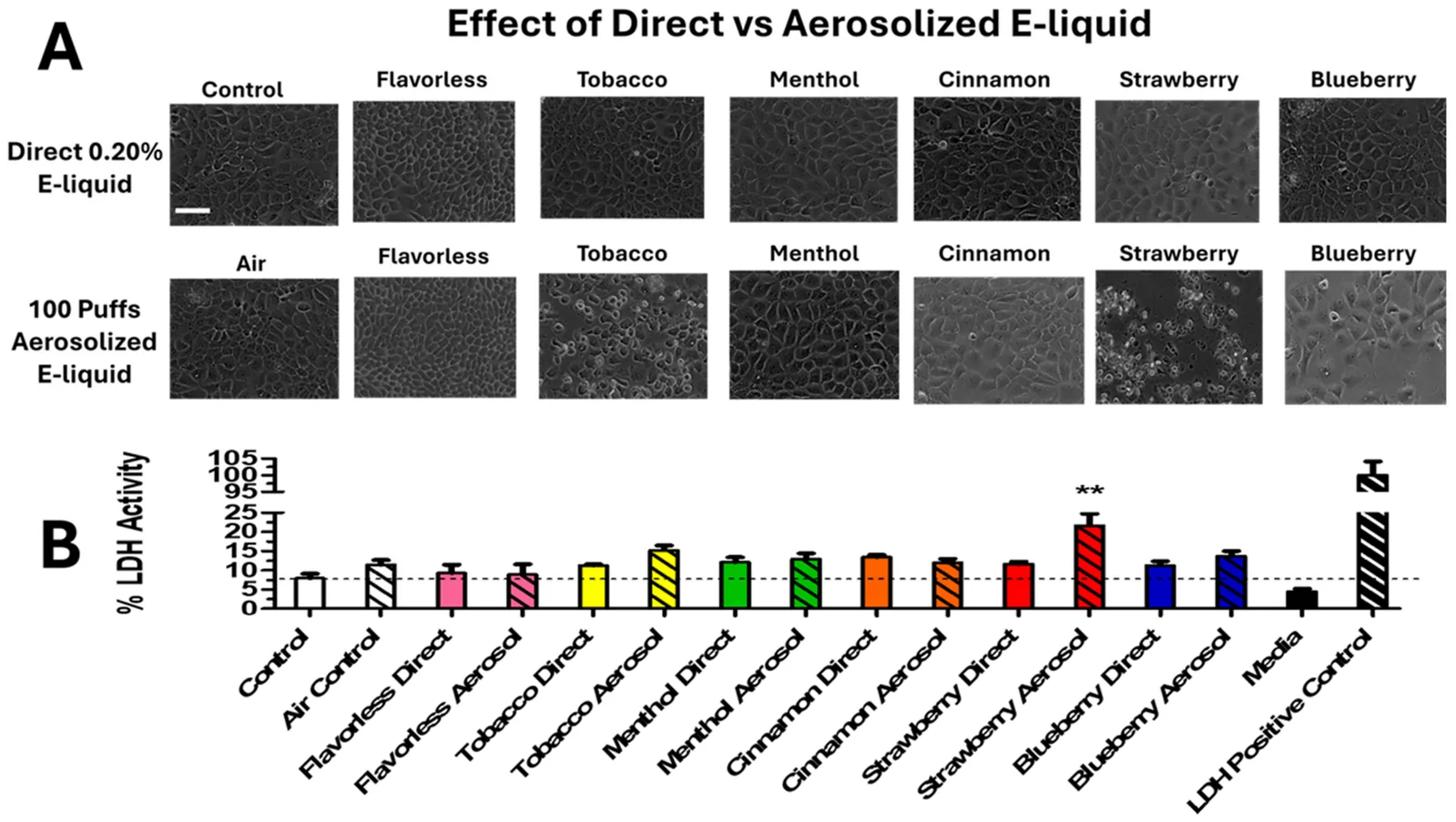
Representative micrographs of OKF6/TERT-2 cells following 24 h incubation in DFK culture media containing either E-liquids ± flavors directly or their bubbled aerosols (**A**) along with their respective viabilities as indexed by the LDH cytotoxicity assay (**B**). The white line in the control micrograph = 250 µm. Each bar represents the mean ± SEM (*n* = 8 to 24 independent). Dashed line indicates control level ** = *p* < 0.01 between direct administration of E-liquid (bars) and aerosolized E-liquid (bars with diagonal lines) bubbled into media using two-way ANOVAs.

Figure 7 illustrates the effects of 24 h incubation of OKF6/TERT-2 cells in culture media containing either E-liquids ± flavors or their aerosols on cell morphology (Figure 7A, see also Supplementary Figure S2) and viability as indexed by % LDH activity (Figure 7B, see also Supplementary Table S1). Direct addition of 0.20% E-liquid ± flavors have no discernable effects on the morphology of OKF6/TERT-2 cells. Similarly, 100 puffs of aerosolized E-liquid ± menthol and cinnamon have no obvious effects on the morphology of OKF6/TERT-2 cells. In contrast, aerosolized tobacco-, blueberry- and especially strawberry-flavored E-liquids appear to compromise the typical morphology of the OKF6/TERT-2 cells. As seen in Figure 7A, strawberry-flavored aerosol bubbled into the culture media significantly alters the morphology of these cells. Furthermore, these results are supported by the significant increase (*p* < 0.001) in % LDH activity between direct E-liquid and bubbled aerosol for the strawberry flavor treatment group (Figure 7B). When compared to the air control, which produced 11.5% LDH activity, the bubbled strawberry aerosol produced 21.7% LDH activity. These results suggest that heating and subsequent aerosolization of E-liquid has the potential to induce or amplify cellular cytotoxicity of some flavored E-liquids, such as the strawberry flavor. Note that aerosolized growth media containing slightly less strawberry-flavored E-liquid (0.17%) increases LDH activity while non-aerosolized growth media containing slightly more strawberry-flavored E-liquid (0.2%) does not. This lends further support to the possibility that heating and vaporization may increase the cytotoxic potential of at least some flavored E-liquids.

### 3.6. Effects of E-Liquid Treatments on Cell Viability and Apoptosis Assessed by Flow Cytometry

**Figure 8:**
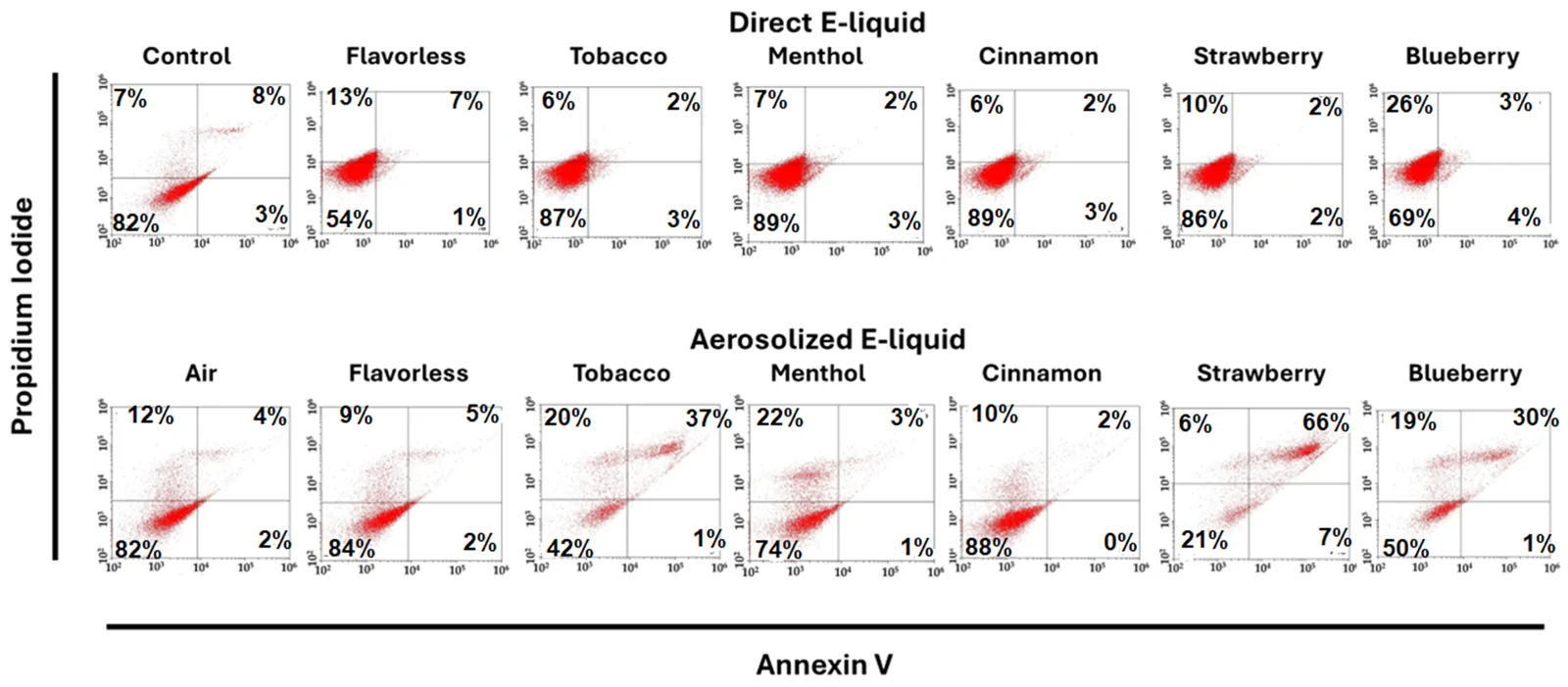
Representative flow cytometry dot plots of OKF6/TERT-2 cells following 24 h incubation in culture media containing either 0.20% direct E-liquids ± flavors or 100 puffs of aerosolized E-liquids bubbled into DFK media. Cells were stained with Annexin V Alexa Fluor™ 488 and PI to distinguish live (Annexin V^−^/PI^−^), necrosis (Annexin V^−^/PI^+^), apoptosis (Annexin V^+^/PI^−^), and apoptosis/necrosis (Annexin V^+^/PI^+^) populations. The numbers within each quadrant, as indicated by the central cross hairs, represent the % distribution of cell populations based on fluorescence intensity. For more detail concerning these dot plots, see Figure 2.

Figure 8 shows representative flow cytometry plots of the effects of direct E-liquids ± flavors and their aerosolized forms on cell viability and cell death in OKF6/TERT-2 cells following 24 h exposure (see Figure 2 for detailed explanation of flow cytometry dot plots and Supplementary Figure S3 for enhanced view). Cells were stained with Annexin V Alexa Fluor™ 488 and PI to differentiate viable (Live) (Annexin V^−^/PI^−^), necrosis (Annexin V^−^/PI^+^), apoptosis (Annexin V^+^/PI^−^), and apoptosis/necrosis (Annexin V^+^/PI^+^) populations. Control and direct E-liquid treatment groups maintained high cell viability, with approximately 85 to 90% of cells classified as live and relatively low percentages of apoptotic and necrotic populations. In contrast, exposure to air and aerosolized E-liquids resulted in a reduction in the percentage of live cells and an increase in necrotic and apoptotic/necrotic populations.

**Figure 9:**
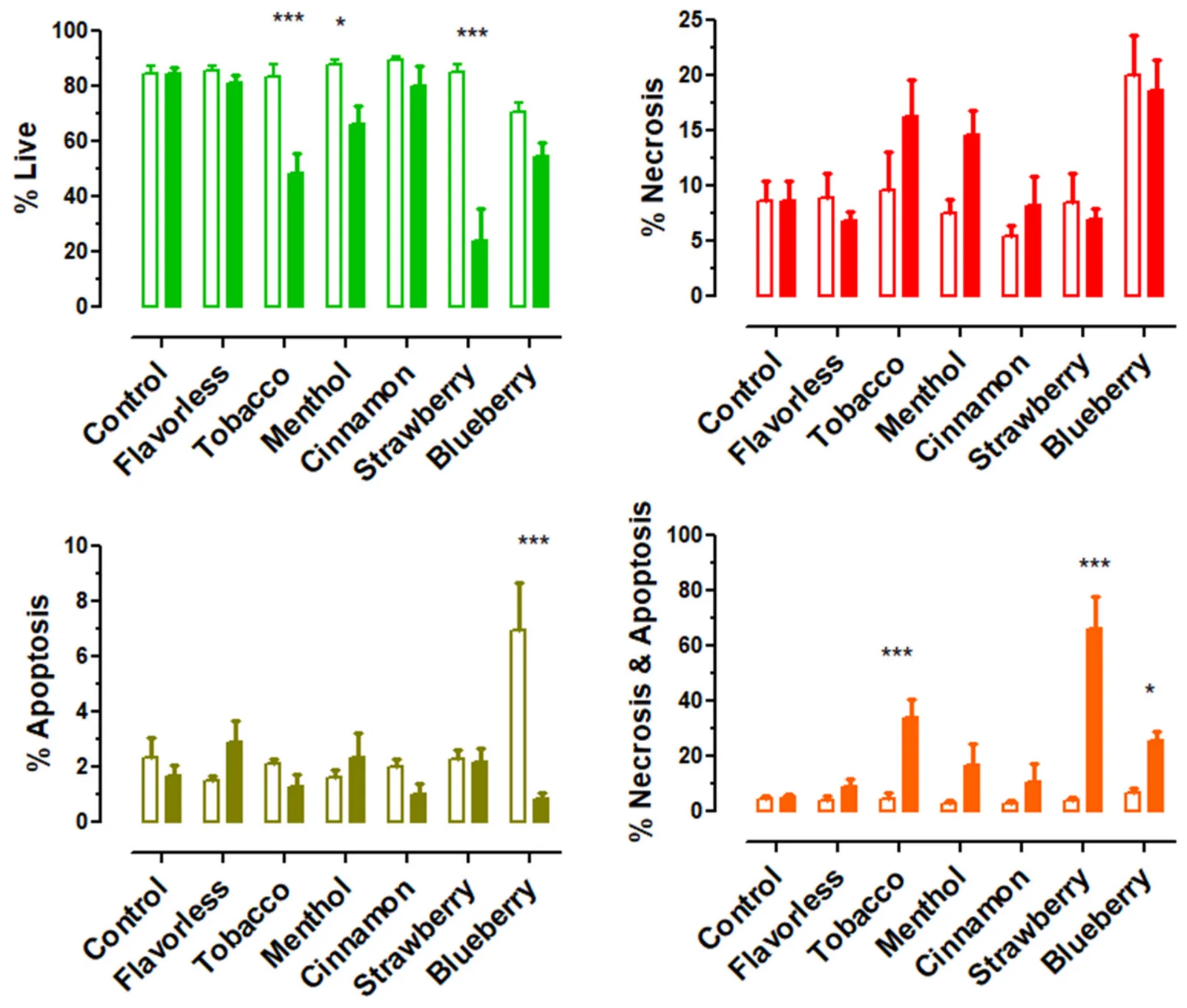
Distribution of cell populations classified as live (**top left**), necrotic (**top right**), apoptotic (**bottom left**), and combined apoptotic/necrotic (**bottom right**) following 24 h exposure to direct E-liquids ± flavors (unfilled bars) or aerosolized E-liquids (filled bars). Each bar represents the mean ± SEM (*n* = 6 to 13 independent samples). Control refers to no E-liquid (unfilled bar) or air (filled bar). * = *p* < 0.05, and *** = *p* < 0.001 between direct administration of E-liquid (unfilled bars) and aerosolized E-liquid (filled bars) bubbled into media using two-way ANOVAs.

Quantification of flow cytometry dot plots are shown in Figure 9 and Table S1 and corroborate the results in Figure 8. Control and control air live-cell populations hover at about 85%. When comparing direct E-liquid treatment with aerosol treatment, strawberry flavored aerosol produces the most drastic effect, reducing the live cell population to approximately 24% (*p* < 0.001) while increasing the necrosis/apoptosis population to approximately 66% (*p* < 0.001). Tobacco and menthol aerosols also reduce cell viability, with live cell populations of approximately 49% (*p* < 0.001) and 66% (*p* < 0.05), respectively, but only tobacco aerosol shows an increase in combined necrosis and apoptosis. Interestingly, treatment with blueberry-flavored E-liquid yields an increase (*p* < 0.001) in the number of apoptotic OKF6/TERT-2 cells while treatment with 100 puffs blueberry-flavored E-liquid aerosol increases (*p* < 0.05) the number of necrotic/apoptotic cells. These observations may reflect the decrease in percent live OKF6/TERT-2 cells when cells are treated with both non-aerosolized and aerosolized blueberry-flavored E-liquid, as compared to control (13.9% decrease) and air control (29.8% decrease), respectively. This indicates a possible interaction between flavor types and exposure types (i.e., non-aerosolized vs. aerosolized).

Overall, these results indicate that aerosolized E-liquids, particularly strawberry, increase cell death in OKF6/TERT-2 cells, primarily through necrosis/apoptosis. These findings are consistent with LDH cytotoxicity results (Figure 7B), which also show increased cell damage following the exposure to aerosolized strawberry E-liquids.

### 3.7. Effects of E-Liquid Treatments on the Percent Area of Nuclei Assessed by DAPI Staining

**Figure 10:**
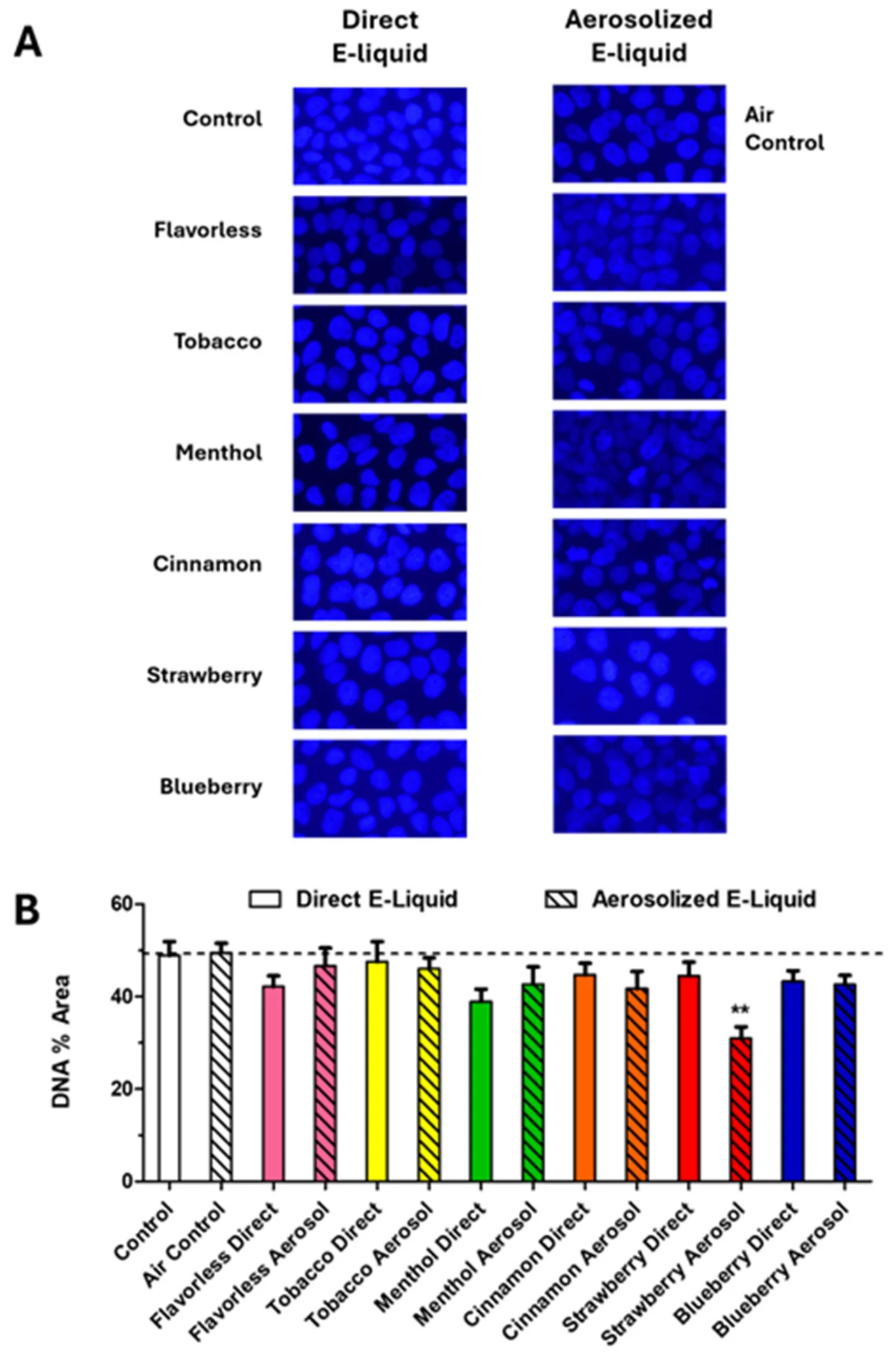
DAPI-determined DNA content of OKF6/TERT-2 cells treated with DFK media containing direct administration of E-liquid ± flavors or 100 puffs of E-liquid ± flavors aerosolized into DFK media. Representative micrographs of OKF6/TERT-2 cell nuclei stained with DAPI are shown in (**A**) and the area of micrographs occupied by OKF6/TERT-2 cell nuclei are shown in (**B**). Photographic images were taken at 400× total magnification (resolution = 1920 × 1080 pixels). Each bar represents the mean ± SEM (*n* = 6 to 11 independent samples). Dashed line indicates control level. ** = *p* < 0.01 between direct administration of E-liquid (unhatched bars) and aerosolized E-liquid (hatched bars) bubbled into media using two-way ANOVAs.

Representative micrographs of DAPI-stained OKF6/TERT-2 cell nuclei are shown in Figure 10. There are no discernable differences in the typical appearance of cell nuclei between treatment groups (Figure 10A), although the percent area of nuclei (Figure 10B and Table S1) in cells treated with 100 puffs of strawberry flavored aerosol is reduced. The nuclei in the control and air control micrographs occupy 48.9 ± 3.0% and 49.9 ± 3.1% area, respectively. The percent area of nuclei for all other treatment groups, except for strawberry flavored aerosol, ranged between 38.9% and 47.6% of the micrographs. In contrast, the percent area of nuclei in cells treated with strawberry-flavored aerosol is significantly (*p* < 0.001) reduced to 31.0 ± 2.5%. These results are consistent with the LDH cytotoxicity data shown in Figure 7B and the flow cytometry data shown in Figure 9.

## 4. Discussion

In this report we describe and use an E-liquid aerosol generating system, which bubbles this material into DFK culture media. Using two 3 s puffs/minute at a flow rate of 1.1 mL/minute [32], and a known weight, volume, concentration of nicotine in E-liquid and the E-liquid specific density (1.18 g/mL) [34], the percent recovery of nicotine (and by extension, E-liquid) is calculated to be 21.3% after 100 puffs, which corresponds to 0.17% aerosolized E-liquid in the DFK (*v*/*v*). The calculations used to determine nicotine percent recovery are outlined in Figure 4 and Table 2. Based on stable temperature readings of the outer surface of the ECIG cartridge (≈30 °C as determined by K-type thermocouple probes and ≈40 °C as determined by FLIR technology; see Supplemental Figure S1), the production of aerosol bubbled into the DFK media remains consistent for at least 60 min. Settings of power, voltage and resistance applied to the coil within the cartridge are 8.8 W, 4.2 V and 2.0 Ω, respectively, which increases the temperature of the coil surrounded by a PG/VG mixture to ≈190 °C [39], which is also the temperature that a PG/VG mixture begins to boil [40].

**Figure 11:**
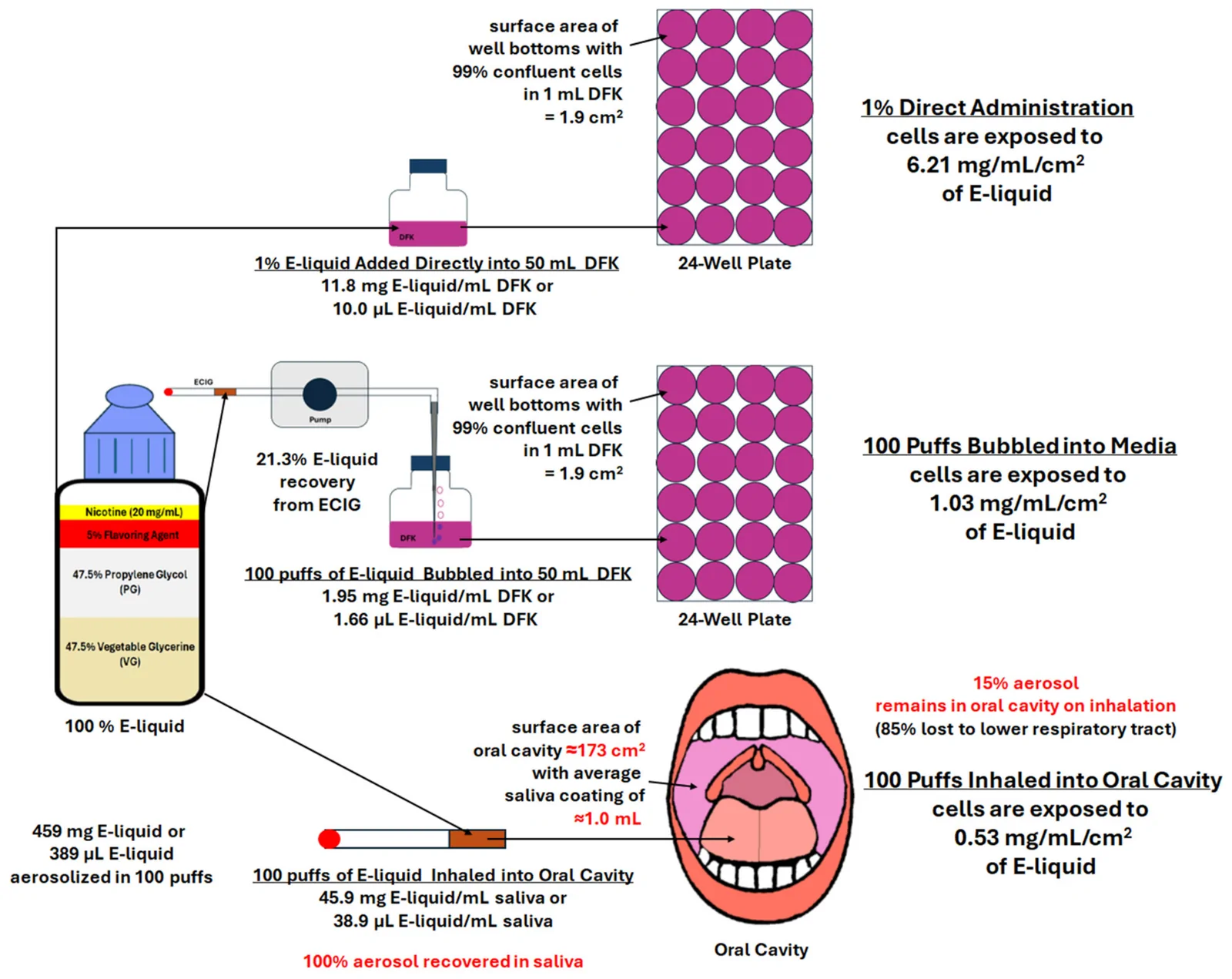
Comparison of OKF6/TERT-2 cells exposed to direct administration of 1% E-liquid into DFK media vs. 100 puffs of E-liquid aerosol bubbled into DFK media vs. 100 puffs of in vivo inhalation of E-liquid aerosol into the oral cavity (hypothetical) as expressed by E-liquid concentration/cm^2^. Text in red indicates assumptions made in calculation.

Using the in vitro conditions outlined above, comparisons of 100 puffs E-liquid aerosol bubbled into media with 1% direct E-liquid administration into media or with more realistic in vivo vaping could be ascertained for oral epithelial cells and is shown in Figure 11. For aerosolized E-liquid bubbled into media, oral epithelial cells are exposed to 1.95 mg E-liquid/mL DFK. If the bottom of a well of a 24-well plate is 1.9 cm^2^, the cells in the well are exposed to 1.03 mg/mL/cm^2^ of aerosol dissolved in the media [41], while oral epithelial cells are exposed to direct administration of 1% E-liquid yields 6.21 mg/mL/cm^2^, a value ≈ 6 times greater.

As indicated in Figure 11, this in vivo theoretical value is based on four assumptions. First, it is assumed that greater than 85% of the E-liquid aerosol is directed toward the lower airways leaving less than 15% of the aerosol in the oral cavity [41,42]. The volume of the oral cavity (based on maximum water capacity of the oral cavity) is determined to be 55.4 mL in females and 71.2 mL in males [41], which represent 11% and 14% of a 500 mL tidal volume, respectively. The second assumption is that the average volume of saliva within the oral cavity is 1 mL [43]. The third assumption is that the area of the oral cavity is ≈173 cm^2^ [44]. The last assumption is that 100% of the aerosolized E-liquid is dissolved in the saliva, although the amount of nicotine, PG and VG absorbed by the oral cavity is highly dependent on aerosol particle size and has been shown to be less than 10% [45,46]. While these assumptions highlight the complexity of aerosol dynamics within the human respiratory tract, it is believed that the in vitro aerosol exposure system used in this study achieves a closer approximation of in vivo vaping.

Based on the calculations of this investigation, the E-liquid aerosol generating system used in this study exposes oral epithelial cells in vitro to 1.03 mg/mL/cm^2^, nearly two times more aerosol (0.53 mg/mL/cm^2^) as compared to in vivo exposure (Figure 11). Given the broad nature of the assumptions used to calculate in vivo aerosol delivery, we speculate that this in vitro vs. in vivo difference may not be significant. Granted, while the ECIG used in this study is a first-generation device (less powerful than current fourth- and fifth-generation devices), and the E-liquid is a simple formulation of PG/VG, flavors, and freebase nicotine (not the highly absorbable salt form), the differences between in vitro and in vivo aerosol exposures should be relatively the same, regardless of the device or E-liquid formulation used. By using this in vitro means of aerosol exposure, more accurate conclusions as to the effects of vaping on oral epithelial cells can be derived. With that said, equating the amount of E-liquid that is directly administered into DFK with the amount of E-liquid aerosol captured by bubbling into the media can reveal if the heating and aerosolization process influences biological effects. In other words, do heating and aerosolization alter the composition of the E-liquid, which in turn alters the biology of the cells exposed? From this investigation, direct administration of 0.17% E-liquid into DFK media is the equivalent of 100 puffs of E-liquid aerosol bubbled into DFK media (Figure 5 and Figure 6). The results of this investigation indicate direct administration of 0.2% E-liquid had no effect on the morphology and viability of OKF6/TERT-2 cells. In contrast, 100 puffs of E-liquid aerosol with some flavored E-liquids, primarily strawberry, induced morphological changes and reduced viability of OKF6/TERT-2 cells. This indicates that chemical alterations may occur from the heating and aerosolization process of some flavored E-liquids.

Previous studies from our laboratory investigated the effects of direct E-liquid administration into DFK culture media [27,28]. Shamim et al. (2025) [28] found 24 h treatment of 0.1% and 0.5% E-liquid ± flavors to have no effect on the morphology of OKF6/TERT-2 cells, while 1% cinnamon-flavored E-liquid impacts cellular confluency and wound healing ability. Cuadra et al. (2023) [27] report that direct administration of 1% E-liquid ± menthol or cinnamon for 24 h significantly increases cytotoxicity of OKF6/TERT-2 cells as indexed by % LDH activity. Like the Shamim study [28], this investigation finds no morphological or cytotoxic effects when OKF6/TERT-2 cells are treated with 0.17% E-liquid for 24 h. In contrast, when these same cells are treated with DFK media previously bubbled with 100 puffs of aerosol, there is a drastic change in the morphological appearance of cells treated with strawberry flavored E-liquid aerosol, which is accompanied by an increase in cellular cytotoxicity as indexed by LDH activity (Figure 7). Furthermore, flow cytometry (Figure 8 and Figure 9) reveals that direct administration 0.17% blueberry flavored E-liquid only slightly decreases (13.9%) the number of live cells while DFK media previously bubbled with 100 puffs of tobacco, strawberry and blueberry flavored aerosols more severely decreases the number of live cells by 35.8%, 60.8% and 29.8%, respectively. This reduction in cell numbers for both direct and bubbled DFK media is primarily attributed to combined necrosis and apoptosis. The cytotoxicity differences shown by LDH activity (Figure 7) and flow cytometry data (Figure 8 and Figure 9) are most likely a function of molecular size where PI is a much smaller molecule than LDH, consequently traversing the compromised cell membrane with greater ease. The percent area of DNA staining with DAPI is reduced only when treated with strawberry flavored aerosol (Figure 10), which supports the reduced number of similarly treated live cells observed in the LDH and flow cytometry assays. However, no distinction in the shape of DAPI stained nuclei between direct E-liquid administration and aerosolized E-liquid treatments could be ascertained. To be clear, a reduction in DAPI staining only suggests a decrease in cell number and does not reflect DNA damage, apoptosis, or fragmentation. These results show a clear distinction between direct strawberry E-liquid administration and aerosolized strawberry E-liquid treatments, suggesting that the heating and aerosolization process contributes to the decreased number of OKF6/TERT-2 cells. This is consistent with the LDH cytotoxicity data (Figure 7) and the flow cytometry data (Figure 9).

Multiple studies have tested the effects of E-liquids on oral epithelial cells and fibroblasts, showing clear toxicity with many flavors, especially menthol or mint [47,48,49,50]. Similarly, ECIG-generated aerosols have been shown to exhibit decreased wound healing ability, cytotoxicity, apoptosis and DNA damage in oral epithelial cells and fibroblasts [51,52,53,54,55,56,57], even though the means by which the aerosols are delivered to the cells vary from study to study. All these studies support our results where concentrations of direct E-liquid lower than 1% in the media, induce little to no effect on the cells exposed. Furthermore, some aerosolized E-liquids ± flavors result in toxicity, although the concentration of the aerosols in the media vary and in many cases is unknown. Silva-Ribeiro et al. (2023) finds around 12 µg/mL of nicotine in the media after 17 to 18 puffs and Phandthong et al. (2023) report 1.9 µg/mL of nicotine in the media after 10 puffs [58,59], whereas in this study, the average nicotine was 11.5, 19.9 and 33.2 µg/mL after 25, 50 and 100 puffs, respectively (Table 2). While all these studies follow CORESTA guidelines [32], different ECIG devices that vary in physical parameters (wattage, voltage, resistance), alternate methods of capturing aerosol, and number of puffs, as well as the cell models exposed to the aerosol, are used. Nonetheless, those studies find that ECIG-generated aerosols result in cellular toxicity [58,59]. To our knowledge this is the first study that attempts to compare the total theoretical amount of nicotine to its actual yield in cell culture media. Furthermore, in this study, the concentration of E-liquid directly added to the media is equivalent to the E-liquid that is recovered from the aerosolization process, assuming that nicotine is aerosolized at the same rate as all other E-liquid components [24,25,26]. Importantly, comparable amounts of E-liquid directly added vs. aerosolized E-liquid delivered into the culture media induced significantly different outcomes on the cell biology of OKF6/TERT-2 cells, especially when flavors are present. This suggests that the aerosolization process could possibly lead to alterations in the chemistry of the E-liquid and the materials that are produced after heating. We speculate that heavy metals including nickel, lead and chromium leached from the ECIG device during the heating process [17] or the production of aldehydes, VOCs and TSNAs [14,15,16,60] in the aerosol are possible culprits for the cytotoxicity observed in this study. In this study, the final concentration of nicotine in the media, whether the flavored E-liquid is directly added or aerosolized, is between 30 and 40 µg/mL after 100 puffs (Figure 6), and none of the flavored E-liquids increased LDH activity, except for the strawberry aerosol (Figure 7). Similarly, Wang et al. (2023) [61] reported that ECIG aerosol condensate, as opposed to cigarette smoke condensate, in their cell culture media results in little to no cytotoxicity, except for the ECIG condensate with mung bean flavor, measuring a 50% inhibitory concentration (IC50) of 89.7 µg/mL nicotine after 72 h of treatment. Regardless of the differences in ECIG-aerosol generation, E-liquid flavors and cell models used, the IC50 results by Wang et al. (2023) [61], which were greater than 40 µg/mL for all aerosols tested, support our % LDH activity results.

Limitations of this study include the use of only one cell type (i.e., OKF6/TERT-2 cells), a limited number of E-liquid flavors (i.e., tobacco, menthol, cinnamon, strawberry and blueberry), the use of a first-generation “cig-a-like” device to generate aerosol and only one time-point post-treatment. Since OKF6/TERT-2 cells are derived from a healthy human donor, these cells have been immortalized and closely represent normal oral epithelial cells. Therefore, these cells were exclusively used in this study since the aim is to determine the effects of flavored aerosols on human oral epithelial cells. The flavors used in this study are the same flavors we have used in previous studies [27,28]. To expand on our research, while maintaining consistency between studies, we have elected to use these same flavors. With that said, these flavors represent a wide range of flavors indicated in the Krüsemann et al. (2019) [62] E-liquid flavor wheel. While newer fourth- and fifth-generation ECIG devices are more powerful and capable of generating more aerosol than the first-generation “cig-a-like” used in this study, we decided to use this ECIG device for two reasons. First, this device is actuated with negative pressure. When the pump is cycled on and off between puffs, enough negative pressure is generated to activate the ECIG while the pump is active. Secondly, “cig-a-like” devices are low power and generate less aerosol at lower temperatures, hence minimizing the leaching of heavy metals and the production of carbonyls, aldehydes and other VOCs induced by heating. Consequently, the results indicate that even low-power devices can generate aerosols that adversely affect OKF6/TERT-2 cell biology. We speculate that these toxins are present in the aerosol, albeit at lower concentrations, and may contribute to chemical alterations in the flavor component of E-liquid aerosol, but we do not know what these alterations entail, nor is it in the scope of this work. Despite that just one time-point was evaluated, the 24 h treatment with aerosols containing tobacco, strawberry and blueberry flavors yielded morphological and cytotoxic aberrations compared to the control.

There is a dire need for further inquiry regarding the effects of vaping on the oral cavity, which represents the first anatomical tissue assaulted by ECIG-generated aerosols. Our lab is currently investigating the effects of E-liquid aerosols ± flavors in culture media on oral epithelial cells in which various physiological processes such as wound healing, oxidative stress, inflammatory response, regulation of mucin, tight junction and defensin genes are monitored. While this data is useful, its interpretations in modeling the all-encompassing effects of E-liquid aerosol ± flavors in vivo are limited. Caution must be used in extrapolating this data to the oral cavity. For instance, bubbling 100 puffs of aerosol directly into culture medium and exposing only a single immortalized cell line at a single time point does not take into account the numerous important physiological features of oral exposure in vivo. These features include, but are not limited to, aerosol deposition, saliva dynamics, mucosal clearance, intermittent exposure, and tissue architecture. Nor does this model replicate actual user puff topography. To gain a better perspective into the effects of vaping in the oral cavity, confirmation experiments involving primary oral epithelial cells in three-dimensional oral mucosal models using repeated or chronic exposure protocols should be considered.

## 5. Conclusions

This study uses nicotine dosimetry to compare E-liquids and their derived aerosols as part of cell culture media. Based on cell morphology, LDH activity, PI/Annexin V and DAPI staining, this work indicates that strawberry flavored aerosol is more toxic than its E-liquid counterpart, suggesting that heating and aerosolization increases the cytotoxic potential of this flavored E-liquid.

## Figures and Tables

**Table 1:** Nicotine standard curve statistics.

Compound	Injected Concentration (µg/mL)	Retention Time (Minutes) *	Measured Concentration (µg/mL) *	% Deviation from Injected Concentration	Area Under Peak *	±95% Confidence Interval
Nicotine	125.0	11.66 ± 0.15	124.87 ± 0.55	0.10	2,741,837 ± 11,878	±57,169
	62.5	11.25 ± 0.12	62.27 ± 0.47	0.37	1,379,811± 10,114	±29,276
	31.3	12.00 ± 0.76	32.41 ± 2.17	3.55	730,259 ± 47,216	±30,052
	15.6	12.11 ± 0.85	15.62 ± 1.99	0.13	364,844 ± 43,351	±35,201
	7.8	13.05 ± 0.83	7.01 ± 0.66	10.13	177,569 ± 14,382	±38,681

Nicotine straight line equation is Y = 21,756 X + 15,283; LOD (µg/mL) = 0.0013; LOQ (µg/mL) = 0.0038; R^2^ =0.9984. * = mean ± SEM where *n* = 3; Limit of detection (LOD) and limit of quantitation (LOQ) were determined based on the calibration curve.

**Table 2:** Calculations for determination of nicotine percent recovery.

Weight of Aerosolized E-Liquid (mg)	Figure 4A
25 puffs	181.9 ± 19.13
50 puffs	285.7 ± 34.80
100 puffs	458.7 ± 23.29
Volume of Aerosolized E-Liquid (mL)	Figure 4B
25 puffs	0.15 ± 0.02
50 puffs	0.25 ± 0.02
100 puffs	0.39 ± 0.01
Volume of Aerosolized E-liquid/Puff (µL)	
25 puffs	6.17 ± 0.65
50 puffs	4.90 ± 0.35
100 puffs	3.89 ± 0.11
Theoretical Nicotine in Aerosolized E-Liquid (µg/Puff) *	
25 puffs	123.3 ± 12.9
50 puffs	98.0 ± 7.0
100 puffs	77.7 ± 2.3
Theoretical Nicotine Delivered to 50 mL DFK (mg) ^@^	
25 puffs	3.083 ± 0.324
50 puffs	4.901 ± 0.351
100 puffs	7.775 ± 0.395
Actual Nicotine Concentration in 50 mL DFK (µg/mL) ^^^	
25 puffs	11.46 ± 0.43
50 puffs	19.92 ± 1.47
100 puffs	33.19 ± 2.22
Actual Nicotine Content Recovered in 50 mL DFK (mg) ^#^	Figure 4C
25 puffs	0.573 ± 0.021
50 puffs	0.996 ± 0.074
100 puffs	1.660 ± 0.111
Nicotine Percent Recovery (%) ^$^	Figure 4D
25 puffs	18.6 ± 0.7
50 puffs	20.3 ± 1.5
100 puffs	21.3 ± 1.4

* Nicotine content in one puff determined from volume of aerosolized E-liquid per puff and the initial nicotine concentration (20,000 µg/mL). ^@^ Nicotine content aerosolized per puff × the number of puffs. ^^^ Determined by HPLC. ^#^ Nicotine concentration (mg/mL) in DFK × 50 mL. ^$^ Actual/theoretical nicotine content in 50 mL DFK. Values given as mean ± SEM where *n* = 3.

## Data Availability

The original contributions presented in this study are included in the article or Supplementary Material. Further inquiries can be directed to the corresponding author.

## Supplementary Materials

The following supporting information can be downloaded at: https://www.mdpi.com/article/10.3390/dj14100656/s1, Table S1: Vaping parameters and raw data. Figure S1: ECIG cartridge temperatures, Figure S2: Magnified view of OKF6/TERT-2 cells, Figure S3: Magnified view of flow cytometry dot-plots, Figure S4: Magnified view of DAPI stained OKF6/TERT-2 cells.
