# Supplementary material for: Effects of E-Liquids and Their Aerosols with and Without Flavors on OKF6/TERT-2 Oral Epithelial Cells: Consequences of Heating and Aerosolization on Cell Viability
Source: Dent J (Basel). 2026 Oct 9;14(10):656. doi: 10.3390/dj14100656 (PMC13703665; doi:10.3390/dj14100656)
Supplement: Supplementary file 1 [file dentistry-14-00656-s001.zip › Figure S1.pdf]

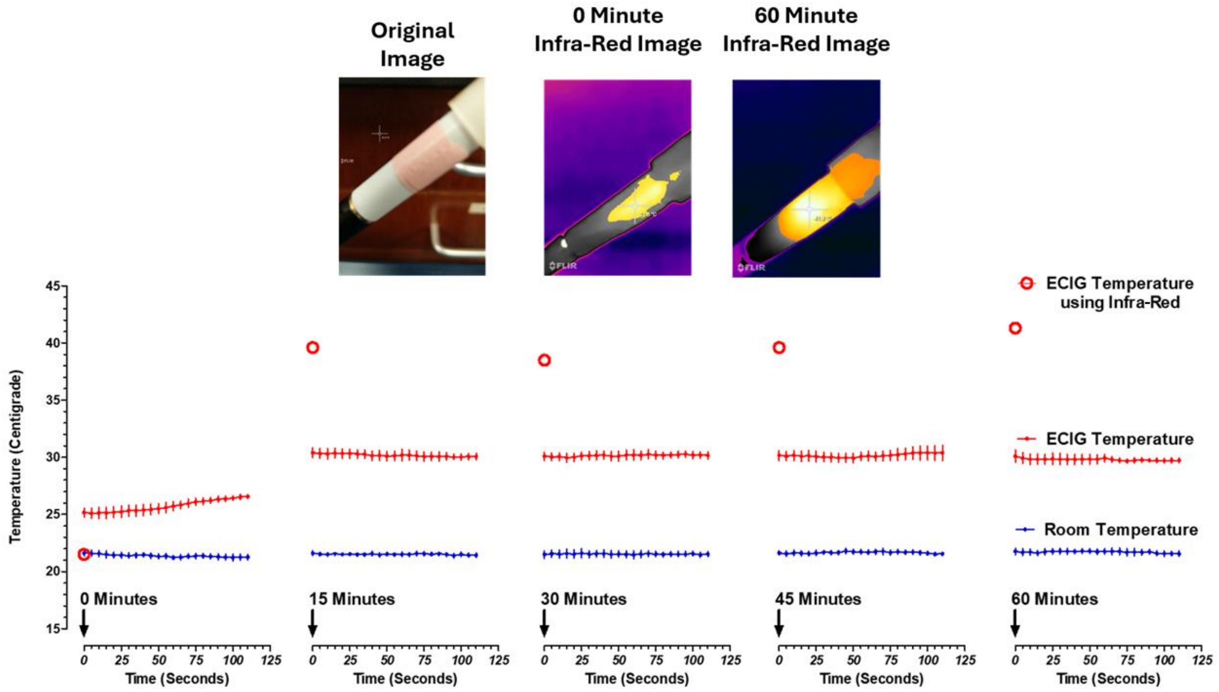

Figure S1: ECIG Cartridge temperature readings using thermocouple temperature probes and infra-red monitoring. A Dickenson (Addison, IL, USA) SM325 data logger equipped with two flexible K-type-thermocouple temperature probes was used. One probe was securely fixed to the ECIG cartridge to monitor its temperature during puffing, and the other probe was left loose to monitor the ambient room temperature. ECIG puff topography consisted of two 3-second puffs/minute at a flow rate of 1.1 mL/minute for 60 minutes. The temperatures of the ECIG cartridge and room air were simultaneously sampled every 5 seconds for 110 seconds beginning at 0, 15, 30, 45 and 60 minutes. Each data point represents the mean ± SEM, n =3. Additionally, ECIG cartridge temperatures were obtained using Forward-Looking Infrared (FLIR) technology available on a Caterpillar (CAT) S62 cell phone (Bullitt Group, Reading, Berkshire, England). Both the temperature of the ECIG cartridge and an infra-red image were obtained at 0, 15, 30, 45 and 60 minutes of the puffing regimen. The 0-minute thermocouple temperatures are higher than room temperature, presumably as a result of a blanketing effect the tape securing the probe to the cartridge has on the temperature. However, the infra-red temperature reading at 0 minutes reflects more closely the thermocouple temperature of room air. While both thermocouple and infra-red temperatures hold steady for up to 60 minutes of puffing, the infra-red temperatures are consistently ≈ 10°C higher than the thermocouple temperatures. The 15-, 30-, 45- and 60-minute infra-red images are similar. Consequently, the images above show only the 0- and 60-minute infra-red images as compared to the original ECIG cartridge image.
