# Supplementary material for: Effects of E-Liquids and Their Aerosols with and Without Flavors on OKF6/TERT-2 Oral Epithelial Cells: Consequences of Heating and Aerosolization on Cell Viability
Source: Dent J (Basel). 2026 Oct 9;14(10):656. doi: 10.3390/dj14100656 (PMC13703665; doi:10.3390/dj14100656)
Supplement: Supplementary file 1 [file dentistry-14-00656-s001.zip › Figure S2.pdf]

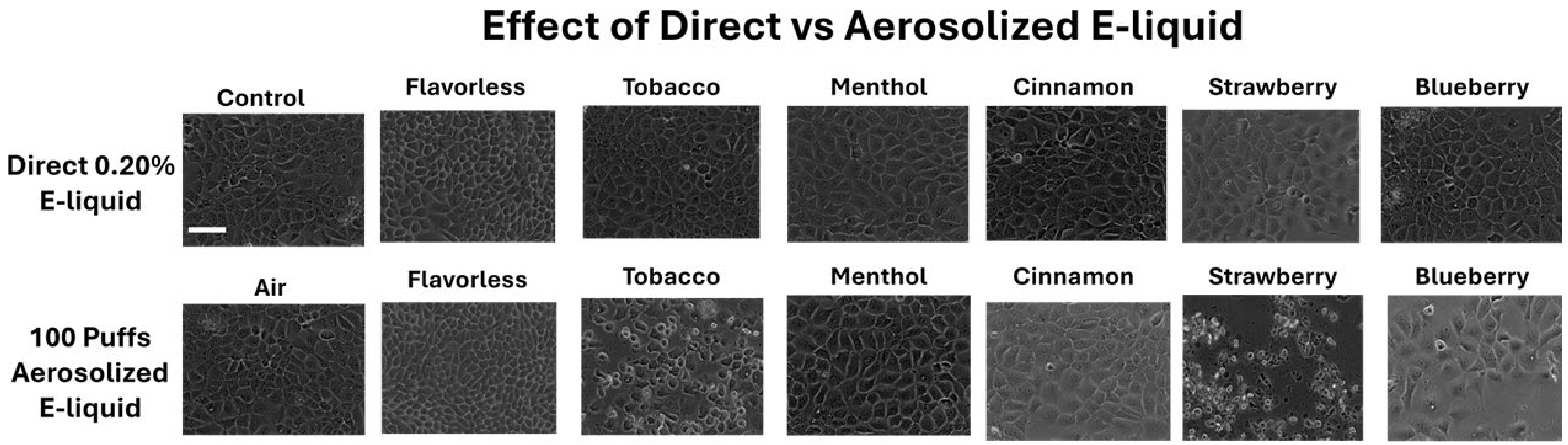

Figure S2: Representative micrographs of OKF6/TERT-2 cells following 24-hour incubation in DFK culture media containing either E-liquids ± flavors directly or their bubbled aerosols. The white line in the control micrograph = 250 μm.
