# Supplementary material for: Effects of E-Liquids and Their Aerosols with and Without Flavors on OKF6/TERT-2 Oral Epithelial Cells: Consequences of Heating and Aerosolization on Cell Viability
Source: Dent J (Basel). 2026 Oct 9;14(10):656. doi: 10.3390/dj14100656 (PMC13703665; doi:10.3390/dj14100656)
Supplement: Supplementary file 1 [file dentistry-14-00656-s001.zip › Figure S3.pdf]

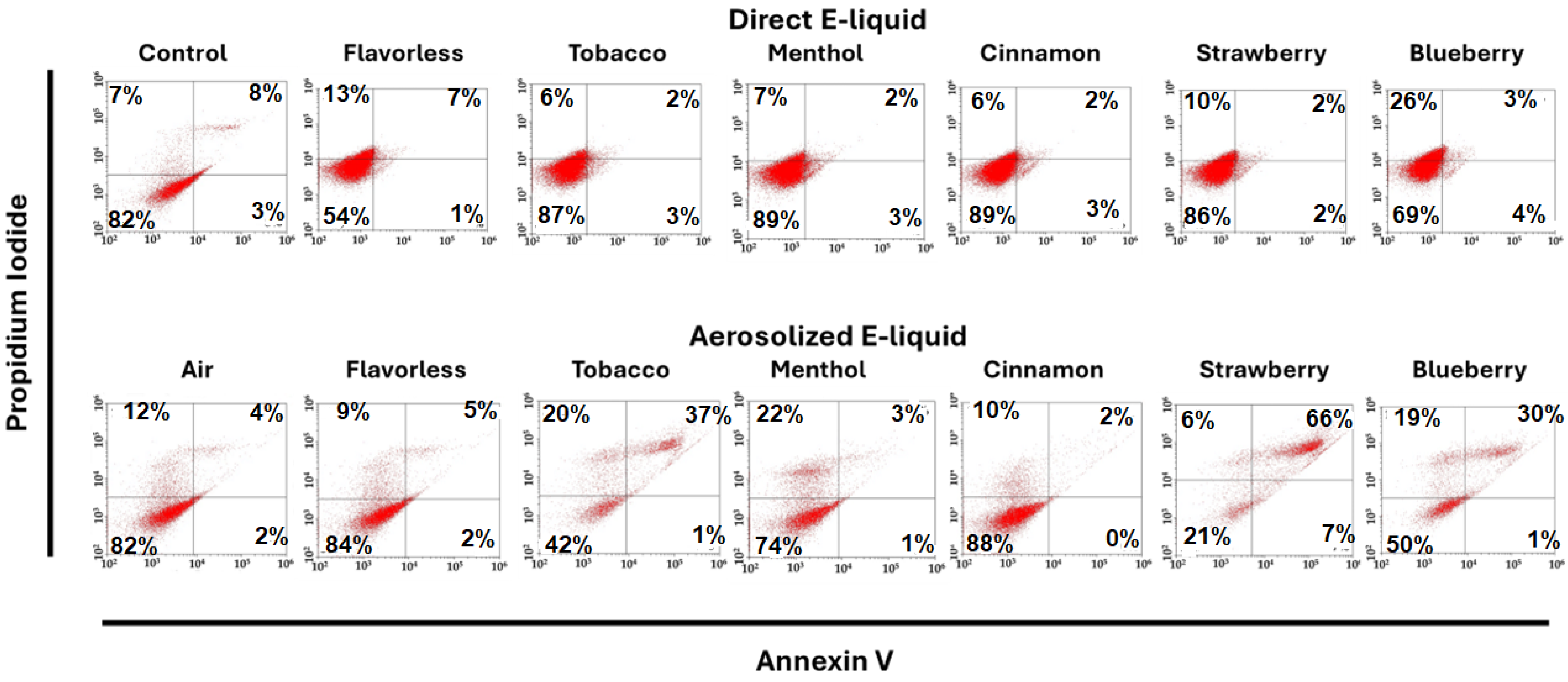

Figure S3: Representative flow cytometry dot plots of OKF6/TERT-2 cells following 24-hour incubation in culture media containing either 0.2% direct E-liquids ± flavors or 100 puffs of aerosolized E-liquids bubbled into DFK media. Cells were stained with Annexin V Alexa Fluor™ 488 and PI to distinguish live (Annexin V^{-}/PI^{-}), necrosis (Annexin V^{-}/PI^{+}), apoptosis (Annexin V^{+}/PI^{-}), and apoptosis/necrosis (Annexin V^{+}/PI^{+}) populations. Quadrants represent the distribution of cell populations based on fluorescence intensity.
