# Supplementary material for: Effects of E-Liquids and Their Aerosols with and Without Flavors on OKF6/TERT-2 Oral Epithelial Cells: Consequences of Heating and Aerosolization on Cell Viability
Source: Dent J (Basel). 2026 Oct 9;14(10):656. doi: 10.3390/dj14100656 (PMC13703665; doi:10.3390/dj14100656)
Supplement: Supplementary file 1 [file dentistry-14-00656-s001.zip › Figure S4.pdf]

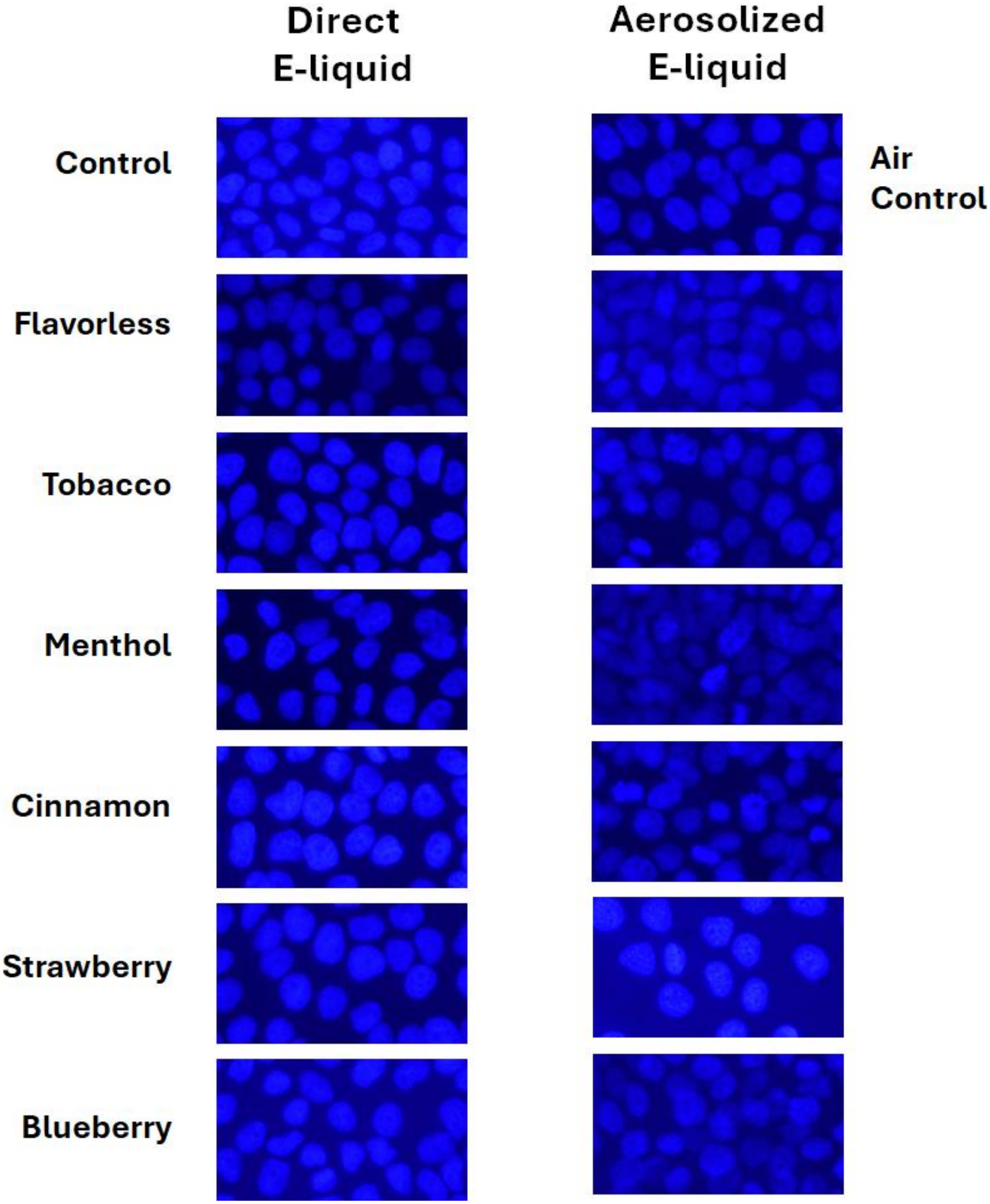

Figure S4: DNA of OKF6/TERT-2 cells treated with DFK media containing 0.2 % E-liquid ± flavors or 100 puffs of E-liquid ± flavors aerosolized into DFK media. Representative micrographs are of OKF6/TERT-2 cell nuclei stained with DAPI.
